# New role of the antidepressant imipramine as a Fascin1 inhibitor in colorectal cancer cells

**DOI:** 10.1038/s12276-020-0389-x

**Published:** 2020-02-20

**Authors:** Begoña Alburquerque-González, Manuel Bernabé-García, Silvia Montoro-García, Ángel Bernabé-García, Priscila Campioni Rodrigues, Javier Ruiz Sanz, Fernando F. López-Calderón, Irene Luque, Francisco José Nicolas, María Luisa Cayuela, Tuula Salo, Horacio Pérez-Sánchez, Pablo Conesa-Zamora

**Affiliations:** 1 Pathology and Histology Department, Facultad de Ciencias de la Salud, UCAM Universidad Católica San Antonio de Murcia, Campus de los Jerónimos, s/n, 30107 Guadalupe, Murcia Spain; 2 Research group “Telomerasa, Envejecimiento y Cáncer,” CIBERehd, Hospital Clínico Universitario Virgen de la Arrixaca, IMIB-Arrixaca, Murcia, Spain; 3 Cell Culture Lab, Facultad de Ciencias de la Salud, UCAM Universidad Católica San Antonio de Murcia, Campus de los Jerónimos, s/n, Guadalupe, 30107 Murcia, Spain; 4 Research group “Regeneración, oncología molecular y TGF-β”, Institute for Biohealth Research from Murcia (IMIB), Hospital Clínico Universitario Virgen de la ArrixacaCarretera Madrid-Cartagena, El Palmar, Spain; 5 Cancer Research and Translational Medicine Research Unit, University of Oulu, Aapistie 5A, FI-90220 Oulu, Finland; 6 Medical Research Center Oulu, Oulu University Hospital, University of Oulu, Oulu, Finland; 7 Department of Physical Chemistry and Institute of Biotechnology, University of Granada, Campus Fuentenueva s/n, 18071 Granada, Spain; 8 Institute of Oral and Maxillofacial Disease, University of Helsinki, Helsinki, Finland; 9 HUSLAB, Department of Pathology, Helsinki University Hospital, Helsinki, Finland; 10 Structural Bioinformatics and High Performance Computing (BIO-HPC) Research Group, Universidad Católica de Murcia (UCAM), Guadalupe, Spain; 11 Clinical Analysis Department, Group of Molecular Pathology and Pharmacogenetics, Institute for Biohealth Research from Murcia (IMIB), Hospital Universitario Santa Lucía, c/Mezquita sn, 30202 Cartagena, Spain; 12 Present Address: C/Mezquita s/n CP, 30202 Cartagena, Murcia Spain

**Keywords:** Colon cancer, Lamellipodia, Drug development

## Abstract

Serrated adenocarcinoma (SAC) is more invasive, has worse outcomes than conventional colorectal carcinoma (CRC), and is characterized by frequent resistance to anti-epidermal growth factor receptor (EGFR) and overexpression of fascin1, a key protein in actin bundling that plays a causative role in tumor invasion and is overexpressed in different cancer types with poor prognosis. In silico screening of 9591 compounds, including 2037 approved by the Food and Drug Administration (FDA), was performed, and selected compounds were analyzed for their fascin1 binding affinity by differential scanning fluorescence. The results were compared with migrastatin as a typical fascin1 inhibitor. In silico screening and differential scanning fluorescence yielded the FDA-approved antidepressant imipramine as the most evident potential fascin1 blocker. Biophysical and different in vitro actin-bundling assays confirm this activity. Subsequent assays investigating lamellipodia formation and migration and invasion of colorectal cancer cells in vitro using 3D human tissue demonstrated anti-fascin1 and anti-invasive activities of imipramine. Furthermore, expression profiling suggests the activity of imipramine on the actin cytoskeleton. Moreover, in vivo studies using a zebrafish invasion model showed that imipramine is tolerated, its anti-invasive and antimetastatic activities are dose-dependent, and it is associated with both constitutive and induced fascin1 expression. This is the first study that demonstrates an antitumoral role of imipramine as a fascin1 inhibitor and constitutes a foundation for a molecular targeted therapy for SAC and other fascin1-overexpressing tumors.

## Colorectal cancer: Antitumor antidepressant

The antidepressant drug imipramine can block the activity of a protein that contributes to the progression of certain aggressive tumors. Serrated adenocarcinoma (SAC) is a form of colorectal cancer with a poor prognosis. A key factor in SAC development is the overexpression of the protein fascin1, which promotes the formation of structures that help cancer cells move around, thereby leading to metastasis. Pablo Conesa-Zamora at Santa Lucia University Hospital in Cartagena, Horacio Pérez-Sánchez at the Universidad Católica de Murcia in Guadalupe, Spain, and coworkers demonstrated that imipramine shows promise in binding to fascin1 and blocking its activity. The team analyzed over 9500 compounds as potential fascin1 blockers, identifying imipramine as a possible option. In tests on human tissues and in vivo studies using zebrafish, the drug reduced cancer invasion and metastasis.

## Introduction

Tumor metastasis is the leading cause of cancer-related deaths^1^. Cell migration and invasion are fundamental features of metastatic cancer cells and involve actin cytoskeleton rearrangement, leading to the formation of protrusive structures, such as filopodia, lamellipodia, and invadopodia, that contribute to cancer cell motility^2^.

Fascin1 is an actin filament (F-actin) bundling protein that plays an important role in the formation of protrusive structures and is poorly expressed or absent in most normal epithelia but upregulated in many human carcinomas, with a crucial role in tumor progression, invasion, and metastasis^3,4^. Numerous studies have implicated fascin1 as a potential therapeutic target and biomarker for aggressive carcinomas^4,5^. Recently, our group identified fascin1 as overexpressed in serrated adenocarcinoma (SAC), a WHO-recognized histological subtype of colorectal carcinoma (CRC), which is characterized by worse prognosis^6^ and a more active invasive front as evidenced by a higher occurrence of tumor budding^7^, E-cadherin loss, and more frequent KRAS or BRAF mutations compared with conventional CRC^8–10^. Migrastatin and its macroketone analogs have been found to efficiently inhibit fascin1, thus decreasing metastatic tumor cell migration, invasion, and further metastasis^1^. However, the complex structure of the macroketone hinders its synthesis, and other anti-fascin1 compounds derived from indazol-furan-carboxamides have been tested^11^. To identify novel molecules targeting fascin1 as potential anti-SAC drugs, we performed an in silico compound library screening and subsequent in vitro and in vivo assays to characterize the anti-fascin1, antimigratory, and anti-invasive potential of the selected compounds.

## Materials and methods

### In silico screening

In silico screening^12^ was applied to propose compounds that might have better fascin1 inhibitory properties than migrastatin. Pharmacophore modeling using LigandScout^13,14^ was employed to the core of the structure of migrastatin (MGS_CORE) so that a ligand-based pharmacophore model^15^ was derived. This model was screened against a subset of the DrugBank library (version 5.0; of 9591 compounds, including 2037 approved by the American FDA, 96 nutraceuticals, and 6000 experimental) after fine-tuning on a high-performance computing (HPC) cluster of all related necessary programs from the LS suite.

### Thermofluor and fluorescence titration

Differential scanning fluorimetry (Thermofluor) and titration assays were performed as described in Supplementary Information S1. Briefly, the thermal denaturation profiles of fascin1 (cat. no. 8411-02, Hypermol, Bielefeld, Germany) were obtained by recording the fluorescence intensity for the FAM, HEX, and T-Red predefined filters. *T*_m_ values were measured as the minimum of the first derivative of the thermal unfolding profile. Changes in *T*_m_ associated with ligand binding were estimated, taking the average *T*_m_ value derived from the free protein internal controls. Drugs were provided by MolPort, Latvia (migrastatin, AnalytiCon Discovery, cat. no. NP-006108, and imipramine, Biotrend Chemicals, BG0219).

### F-actin bundling assay

The actin-bundling activity in the presence of drugs was measured by a low-speed centrifugation assay using the Actin Binding Protein Biochem Kit^TM^ Muscle Actin (Cytoskeleton, Inc., cat. no. BK001) and following the manufacturer’s manual. Additional information is provided as Supplementary Information S1.

### Transmission electron microscopy

Transmission electron microscopy (TEM) was assessed following the methodology of a previous paper^16^. Protocol details are provided as Supplementary Information S1.

### Cell culture

A total of eight human colorectal adenocarcinoma cell lines—SW-480 (CLS Cat# 300302/p716_SW-480, RRID: CVCL_0546), DLD-1 (CLS Cat# 300220/p23208_DLD-1, RRID: CVCL_0248), HCT-15 (CLS Cat# 300229/p23303_HCT-15.html, RRID: CVCL_0292), HCT-116 (CLS Cat# 300195/p19841_HCT116.html, RRID: CVCL_0291), HT-29 (NCI-DTP Cat# HT-29, RRID: CVCL_0320), LS174T (CLS Cat# 300392/p720_LS-174T, RRID: CVCL_1384), SW-620 SW620 (NCI-DTP Cat# SW-620, RRID: CVCL_0547) and LoVo (NCI-DTP Cat# LOVO, RRID: CVCL_0399)—were obtained from the American Type Culture Collection (ATCC, Rockville, Maryland). The cell lines were cultivated at 37 °C in high-glucose Dulbecco’s modified Eagle’s medium (DMEM) containing 10% heat-inactivated fetal bovine serum (FBS), 50 U/mL penicillin, and 50 µg/mL streptomycin (Sigma-Aldrich Chemical Co., USA) in an atmosphere of 5% CO_2_ and 95% humidified air. Subculture was performed when 90% confluence was reached. Human cell line identification by short tandem repeat profile testing, according to the American National Standards Institute, has shown an appropriate match for HCT-116 and DLD-1 cell lines.

### Quantification PCR for fascin1 mRNA expression

Fascin1 gene expression was measured following the protocol previously described^2^. The relative quantitation was obtained by the 2-ΔCt method using β-actin as the housekeeping gene. The amounts of mRNA are given as the number of copies per million copies of β-actin. Primers for β-actin used for FSCN quantitation are shown in Supplementary Information S1.

### Cell viability assay

Exponentially growing cells were plated in triplicate in flat-bottomed 96-well plates (Nunc, Roskilde, Denmark) at 1500 cells/well. On the day after plating, drugs were added in serial dilutions from 500 nm to 300 µM. Control wells contained medium without drug plus 0.1% dimethyl sulfoxide (DMSO) (drug carrier). The plates were incubated for 3 days in a humidified 5% CO_2_ incubator and assayed for cell viability. Tetrazolium, dissolved in phosphate-buffered saline, pH 7.2, at 1.9 mg/mL was added to the cells (30 µL/well). Additional information is provided as Supplementary Information S1.

### RNA labeling, microarray hybridization, and functional enrichment analysis

To obtain a general overview of enriched functions associated with the treatment of colorectal cancer tumor cells, HCT-116 cells were treated with DMSO or imipramine. Detailed information is included in Supplementary Information S1. Datasets are deposited in the Gene Expression Omnibus database under accession number GSE125169.

### Immunofluorescence

For the immunofluorescence assays, round coverslips (Thermo Fisher, Waltham, Massachusetts) were seeded with HCT-116 cells in the presence of 10% FBS. Artificial wounding was performed by transversally dragging a sterilized razor blade on the central area of the coverslips. Briefly, cancer cells were treated with 100 µM migrastatin, 20 µM imipramine, 10 ng/mL epidermal growth factor (EGF), or 50 μM MEK inhibitor PD98059 (MEKi) (both from Sigma-Aldrich, St. Louis, Missouri, USA) for 24 h. Additional information is shown as Supplementary Information S1.

### Cell migration assay

Cell migration was executed with SW-480, DLD-1, and HCT-116 cell lines by performing the wound-healing assay in the presence of 10% FBS. Colorectal cancer cells (50,000 cells) were plated in low 35-mm dishes with culture inserts (Ibidi, Martinsried, Germany). After appropriate cell attachment and monolayer formation (approximately 24 h), inserts were then removed with sterile forceps to create a wound field of approximately 500 µm, according to the manufacturer’s protocol. Additional information is shown in Supplementary Information S1.

### Transwell invasion assay

The invasive capacities of HCT-116 cells were determined using a Cell Biolabs Cytoselect^TM^ 24 Well Cell Invasion Assay Kit (Basement Membrane Colorimetric Format, Cat: CBA-110, Cell Biolabs, CliniScience, Barcelona, Spain) with coated transwell chambers (8 μm pore size) following the instruction manual. Cells were resuspended in serum-free medium and treated under the corresponding conditions (0.1% DMSO, 100 µM migrastatin, and 20 µM imipramine). Additional information is shown in Supplementary Information S1.

### Myoma organotypic invasion model

Cancer cell invasion was assessed in the myoma organotypic cultures and performed according to the previously published myoma model protocol^17,18^. Briefly, uterine leiomyoma tissues were obtained from routine surgery after informed consent of the donors. Myoma (8 mm disks) were preincubated with migrastatin (100 μM), imipramine (10 µM and 20 µM), or 0.1% DMSO-DMEM treatments at 4 °C for 48 h and placed into transwell inserts (6.5 mm diameter; Corning Incorporated, Corning, New York), and 700,000 cells were added on top of each myoma disk. The cells were allowed to attach overnight on the myoma disks, transferred onto uncoated nylon disks, and treated with the compounds for 14 days. After fixation, 6-μm sections were cut and stained with cytokeratin AE1/AE3 (M3515, Dako). ImageJ v1.46o was used to measure invasion areas and depths. Each treatment was performed in triplicate. Additional information is shown in Supplementary Information S1.

### Transfection assay

The day before transfection, DLD-1 cells were seeded in six-well plates and then cultured overnight. The transfection efficiency of lipofectamine 2000-based liposomes was determined using the GFP reporter gene. The pGFP-N3 control vector (MOCK) and fascin1-GFP vector were kindly provided by Dr. Milind Vaidya from the Advanced Centre for Treatment Research and Education in Cancer (Maharashtra, India). The final amount of DNA plasmids was 1 µg/well. Details regarding this experiment are provided as Supplementary Information S1.

### Zebrafish invasion and metastasis assays

The colonization of zebrafish (*Danio rerio*) embryos by human cancer cells was performed as previously described^19^. Trypsinized, washed colorectal cancer cells were stained with fluorescent CM-Dil (Vybrant, Invitrogen), and 50–100 labeled cells were injected into the yolk sac of dechorionated zebrafish embryos. The viability of zebrafish embryos was assessed under 100 μM migrastatin or imipramine (5, 10, and 20 µM) treatments and under the combined effect of compound treatment and tumor cell injection. The evaluation criterion for embryos being colonized by human cancer cells was the presence of more than three cells outside the yolk sac. The metastasis assay was based on previous works by Fior et al.^20^, and a metastatic potential assay on zebrafish was performed. Transfected DLD-1-overexpressing fascin1 and native HCT-116 cells were stained and xenografted as already mentioned. From the third day post injection, larvae were fed with ZEBRAFEED by Sparos (<100 µm) and treated with imipramine daily. At day 6 post injection, larvae were examined for monitoring of tumor growth and invasion using a fluorescence microscope. The evaluation for metastasis potential by human cancer cells was the presence of cell colonies (dividing cells) outside the yolk sac. Fishes with fluorescently labeled cells appearing outside the implantation area at 2 h post injection were excluded from further analysis. All the fish were incubated at 35 °C and analyzed with a SteReo Lumar V12 stereomicroscope equipped with an AxioCam MR5 camera (Carl Zeiss). The percentage of invasion and the presence of cell colonies were calculated by the researcher (M.B.-G.) without previous knowledge of the experimental treatment conditions. The experiments were completed in triplicate, obtaining an average value at 4 days post xenograft (invasion assay) and 6 days post xenograft (metastasis assay).

### Chemical treatment

The effect of treatments on the zebrafish invasion model was tested in two ways: one treating cancer cells before injection in the larvae, and the other treating the larvae with the drugs once injected with tumor cells. Additional information is shown in Supplementary Information S1.

### Statistics

Data are expressed as the mean ± standard deviation (SD). The data were analyzed for significant differences by Student’s *t* test for paired and unpaired data after testing for normal distribution of the data. For in vitro experiments, one-way analysis of variance (ANOVA) was performed, followed by a Tukey post hoc test to compare each group. Differences were considered significant at an error probability of *p* < 0.05. SPSS 18.0 software was used for the rest of the statistical analyses (SPSS, Inc., Chicago, Illinois, USA).

### Ethics statement

For the myoma organotypic invasion model, uterine leiomyoma tissues were obtained from routine surgery after informed consent of the donors and their use approved by the Ethics Committee of the Oulu University Hospital, Oulu, Finland, in accordance with the ethical standards laid down in the 1964 Declaration of Helsinki and later amendments. The studies on zebrafish were approved by the Bioethical Committee of the University Hospital Virgen de la Arrixaca, Murcia, Spain.

## Results

### In silico screening identifies imipramine as a plausible ligand for fascin1

**Fig. 1:**
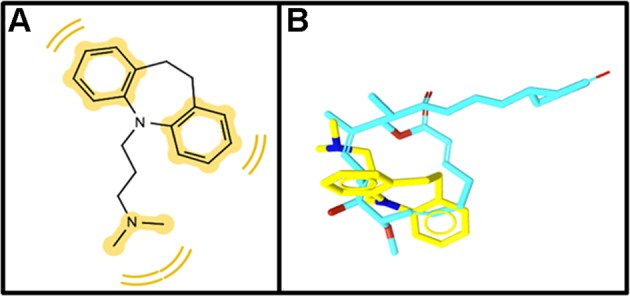
Results obtained after ligand-based pharmacophore screening with LigandScout. **a** 2D diagram of matching hydrophobic pharmacophoric features between (yellow spheres) migrastatin and imipramine. **b** 3D alignment of migrastatin (blue skeleton) and imipramine (yellow skeleton).

In silico screening calculations and careful visual inspection of the results^1,21^ detected imipramine as one of the top 31 candidates for posterior experimental validation. The pharmacophore model derived for imipramine is depicted in Fig. 1.

### Thermofluor and fluorescence titration experiments validate imipramine binding to fascin1 in vitro

**Fig. 2:**
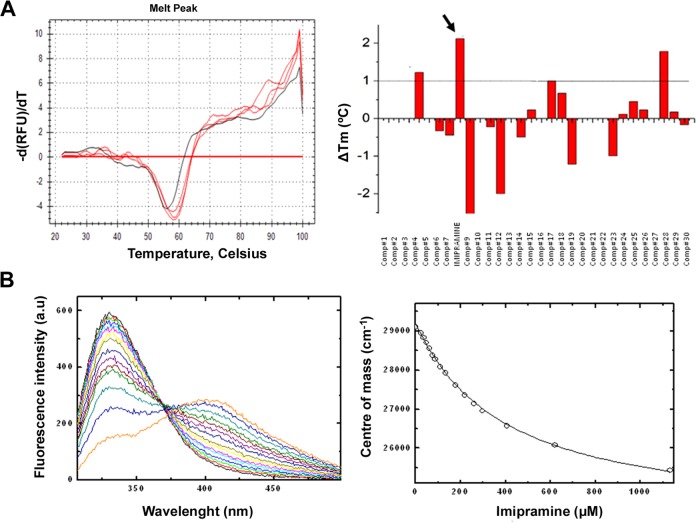
In vitro validation of imipramine binding to fascin1. **a** Differential scanning fluorimetry assays. The right panel shows the first derivative of the thermal denaturation profiles of fascin1 in the absence (black) and in the presence of 1 mM imipramine (red in triplicate). The left panel summarizes the changes in *T*_m_ induced by the presence of the selected compounds at 1 mM in 10% DMSO. **b** Fluorescence titration. The right panel shows the fluorescence emission spectra of fascin1 in the presence of increasing concentrations of imipramine. The left panel shows the binding isotherm. White circles represent the center of mass of the different spectra, and the continuous lines symbolize the best fit of the data to a one-site binding model.

The binding capacity of the distinct compounds to fascin1 was tested using differential scanning fluorimetry. As shown in Supplementary Information S2, at pH 7.4, fascin1 unfolds in a single transition, showing no concentration dependency (panel A) and good tolerance to DMSO (panel B). Average *T*_m_ values for unbound fascin1 were obtained for each internal filter (FAM, HEX, and T-Red) as reference for the determination of the changes in *T*_m_ upon compound binding (*T*_m,FAM_ = 55.6 ± 0.5 °C, *T*_m,HEX_ = 56.0 ± 0.0 °C, *T*_m,Tred_ = 56.2 ± 0.6 °C, where the error values correspond to the SD for the seven replicas included in each plate as internal controls). The results of the thermal shift assay with the top 31 compounds are summarized in Supplementary Information S1 (Table S1) and shown in Fig. 2a. Some compounds resulted in distorted thermal unfolding profiles from which reliable *T*_m_ values could not be extracted, probably due to compound interference with the SYPRO fluorescence signal. Within those that could be analyzed, imipramine resulted in the highest significant increase in *T*_m_ of approximately 2 °C, suggesting specific binding to fascin1 (Fig. 2a, arrow). The binding of imipramine to fascin1 was further validated in vitro using fluorescence titration experiments, rendering a dissociation constant of 390 μM (Fig. 2b).

### Imipramine significantly decreases the fascin1-induced bundles

**Fig. 3:**
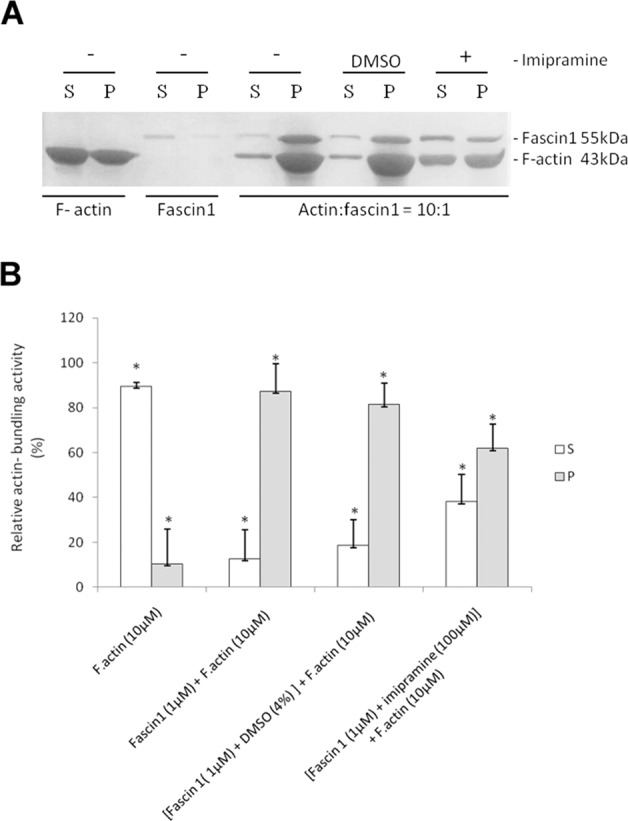
F-actin bundling activity assay. **a** Assay of actin-bundling activity with a low-speed cosedimentation assay. Polymerized F-actin (10 µM) was incubated with fascin1 (1 µM) in the presence (+) or absence (−) of imipramine and DMSO (control). **b** Quantification of the F-actin bundling assay from (**a**). The results are the means and SD (*n* = 4, *p < 0.01).

In the absence of fascin1, although the bundles of F-actin polymers are present in the pellets, the majority of F-actin was observed in the supernatant fraction. As shown in Fig. 3, purified fascin1 increased the amount of F-actin localized in the pellets. However, upon imipramine addition, the F-actin pellet fraction significantly diminished in favor of the supernatant. Taken together, these results indicate that imipramine partially interferes with fascin1 in bundling F-actin polymers.

### Electron microscopy confirms imipramine-driven alteration of actin bundling

**Fig. 4:**
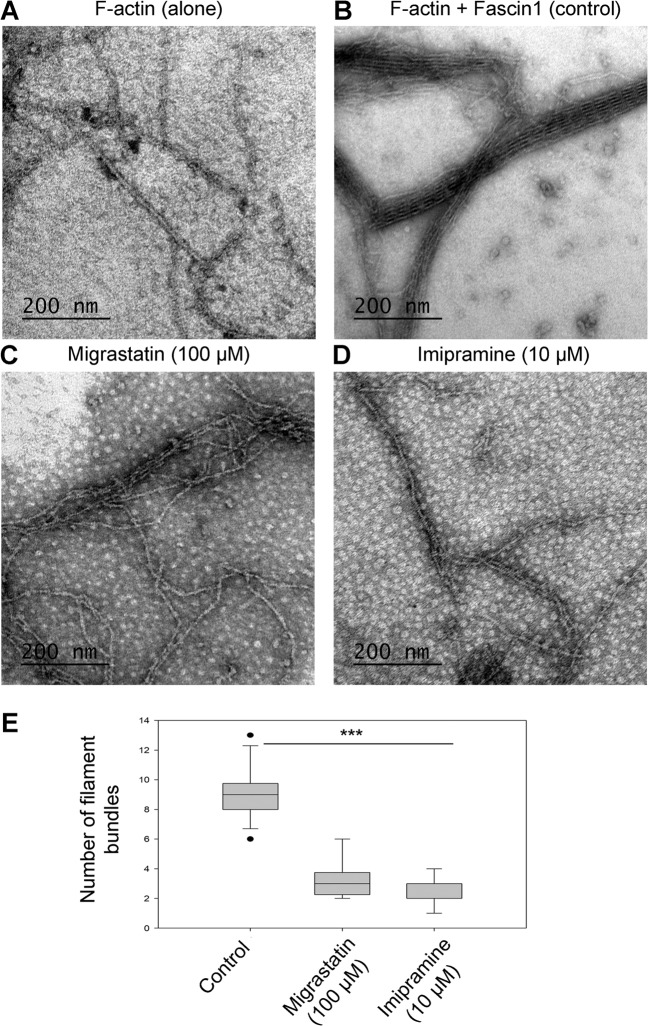
TEM visualization of actin binding and bundling activities in the presence of fascin1 and inhibitors (negative staining). **a** Stained polymerized F-actin filaments alone. **b** Under control conditions (DMSO), actin filaments were incorporated into bundles in the presence of untreated fascin1 at a 1:1 ratio. **c** Binding and bundling assays with filamentous F-actin and fascin1 previously incubated with 100 μM migrastatin. **d** Binding and bundling assays with filamentous F-actin and fascin1 previously incubated with 10 μM imipramine. High-magnification images (×93,000) show examples of aberrant morphology in the treated fascin1 conditions (**c**, **d**). **e** Quantitative analysis of the numbers of actin filaments in the presence of the compounds and comparison to control conditions (***p < 0.001).

Transmission electron microscopy showed that only polymerized F-actin incubated in the presence of fascin1 was able to form filament bundles (9.00 [8.00–9.75]) (Fig. 4). Fascin1 preincubated with 100 μM migrastatin or 10 μM imipramine led to the disorganization of the bundles, resulting in fewer filaments than in control conditions (Kruskal–Wallis test, *p* < 0.001). No significant differences were found between the distinct treatments (Mann–Whitney test, *p* = 0.370).

### The expression of fascin1 mRNA varies among different colorectal cell lines

To choose colorectal cell lines with the highest and lowest endogenous fascin1 expression, RT-qPCR was performed on RNA extracted from the eight colorectal cancer cell lines. Supplementary Information S3 illustrates how HCT-116 and SW-480 cells show the highest fascin1 expression, while LoVo, DLD-1, and HT-29 cells show the lowest expression. Given the ease of cell culture and the suitable morphology with prominent cytoplasm for immunofluorescence, migration, and invasion assessment, DLD-1, SW-480, and HCT-116 cell lines were selected for subsequent in vitro assays.

### High imipramine concentrations compromise cancer cell viability

Compounds might be toxic to cancer cells, and the effects observed could be a consequence of a general effect of cell functionality and not a specific drug effect. For this purpose, a viability assay on DLD-1, HCT-116, and SW-480 cells was performed to set up the working concentration of the drugs. According to data presented as Supplementary Information S4, HCT-116 was more sensitive than DLD-1 and SW-480, and the working concentrations were set up for subsequent in vitro studies at 10 and 20 µM imipramine and 100 µM migrastatin.

### Identification of imipramine-associated enriched functions

To determine which general functions are affected upon imipramine treatment, expression profiling of imipramine-treated HCT-116 colorectal cancer cells from a migration assay was undertaken. Functional enrichment analysis revealed a large number of differentially expressed genes compared with the control treatment (DMSO), among which we selected the top 2000 genes. The Panther Classification System Program was used, and as shown in Supplementary Information S5, 18 Gene Ontology molecular functions were related to these genes. Interestingly, actin binding (GO: 0003779), cytoskeletal protein binding (GO: 0008092), and structural constituent of cytoskeleton (GO: 0005200) were among these 18 functions.

### Imipramine affects fascin1 localization and lamellipodium formation

**Fig. 5:**
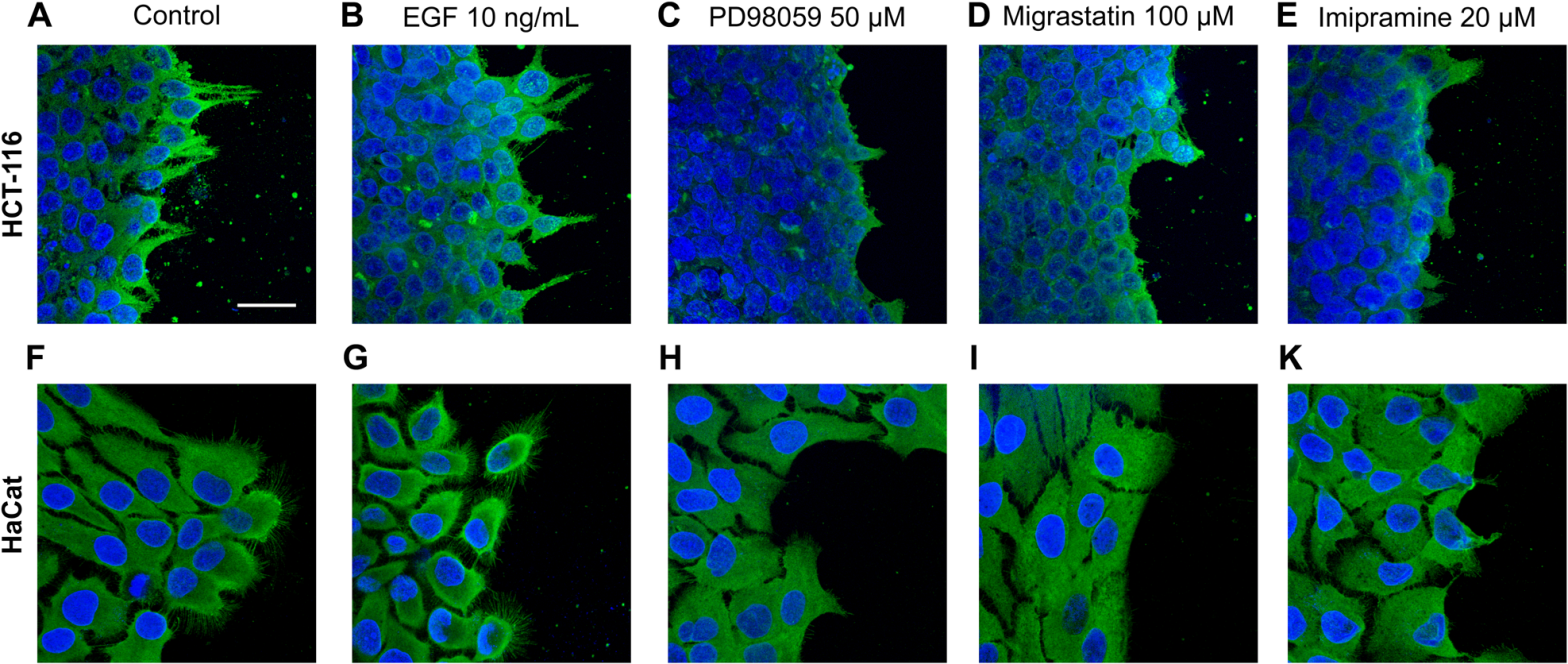
Cell morphology of HCT-116 and HaCat cells upon inhibition of lamellipodia and filopodia formation. Inset shows the immunofluorescence analysis of the fascin1 marker (green) under control conditions: **a**–**f** control conditions, **b**–**g** 10 ng/mL EGF (migration stimulator), **c**–**h** 50 µM PD98059 (MEK inhibitor), **d**–**i** 100 µM migrastatin, and **e**–**k** 20 µM imipramine. Images were captured using an LSM 510 META confocal fluorescence microscope with a ×63 oil objective. Scale bar 30 µm. Migrastatin and imipramine inhibit lamellipodia protrusion and fascin1 localization in a similar way to the migration MEK inhibitor PD98059 in both cell lines.

The effect of imipramine on fascin1 localization and actin reorganization, including lamellipodium formation, was assessed by immunofluorescence. Figure 5 shows the fascin1 localization (green) in two cell lines, the cell line expressing higher fascin1 level, HCT-116, and a nontumoral cell line derived from keratinocyte cells, HaCaT, which was used for illustrative purposes because of its ample cytoplasm. A prominent filopodia and lamellipodium formation associated with fascin1 localization was observed in control conditions and EGF-treated cells, whereas these cytoskeleton structures were absent in those cells treated with migrastatin or imipramine to a similar extent to what was observed with the migration inhibitor targeting the MEK pathway (PD98059). The lamellipodia protrusion number was calculated in HCT-116 cells in a quantitative manner under the different conditions, and this number was significantly lower after migrastatin and imipramine treatments (Supplementary Information S2). Taken together, these results identify an effect of imipramine in diminishing invasive actin cytoskeleton structures and fascin1 localization in such structures.

### Imipramine diminishes the migration of colorectal cancer cells

To explore whether the observed effect of fascin1 inhibitors on lamellipodium protrusion has a reflexive effect on basal cell migration, migrastatin- and imipramine-treated cells were investigated for their migration activity within the linear growth phase (4−7 h after in vitro scratch). As shown in Supplementary Information S6, imipramine produces a remarkable inhibition of migration in all assayed cell lines (*p* < 0.05). Except for SW-480, the imipramine effect was more pronounced than migrastatin, especially for DLD-1. Table S3 shows the quantitative reduction in migration-associated migrastatin and imipramine treatment in DLD-1, SW-480, and HCT-116 colorectal cancer cell lines (Supplementary Information S1). To correlate fascin1 expression with a quantifiable effect on cell migration, fascin1 expression was then silenced in HCT-116 cells and overexpressed in DLD-1 cells (Supplementary Information S1 and S7). Fascin1-silenced HCT-116 cells led to a slight decrease in migration compared with MOCK HCT-116 cells, while double inhibition (epigenetic and pharmacological) produced a significant decrease in migration. Transfected DLD-1 cells overexpressing fascin1 showed a 15% higher migration activity, which was counteracted in the presence of 10 µM imipramine.

### Imipramine inhibits cell invasion through Matrigel

In addition to migration, tumoral cell invasion involves the acquisition of the ability to degrade the extracellular matrix (ECM). For this purpose, a transwell assay was performed on Matrigel, which resembles the ECM composition. As shown in Supplementary Information S8, both migrastatin and imipramine inhibited tumor cell invasion of HCT-116 cells. Similar to the in vitro migration test, the effect of imipramine was slightly more evident than that of migrastatin. Importantly, the addition of imipramine to fascin1 knockdown HCT-116 cells did not decrease invasion further; however, it obviously ameliorated invasion caused by fascin1 overexpression in transfected DLD-1 cells (Supplementary Information S7). This inhibition clearly revealed a fascin1-dependent effect on migration and invasion capacities.

### Imipramine diminishes HCT-116 colon cancer cell invasion in human benign leiomyoma tissue in an in vitro 3D model

**Fig. 6:**
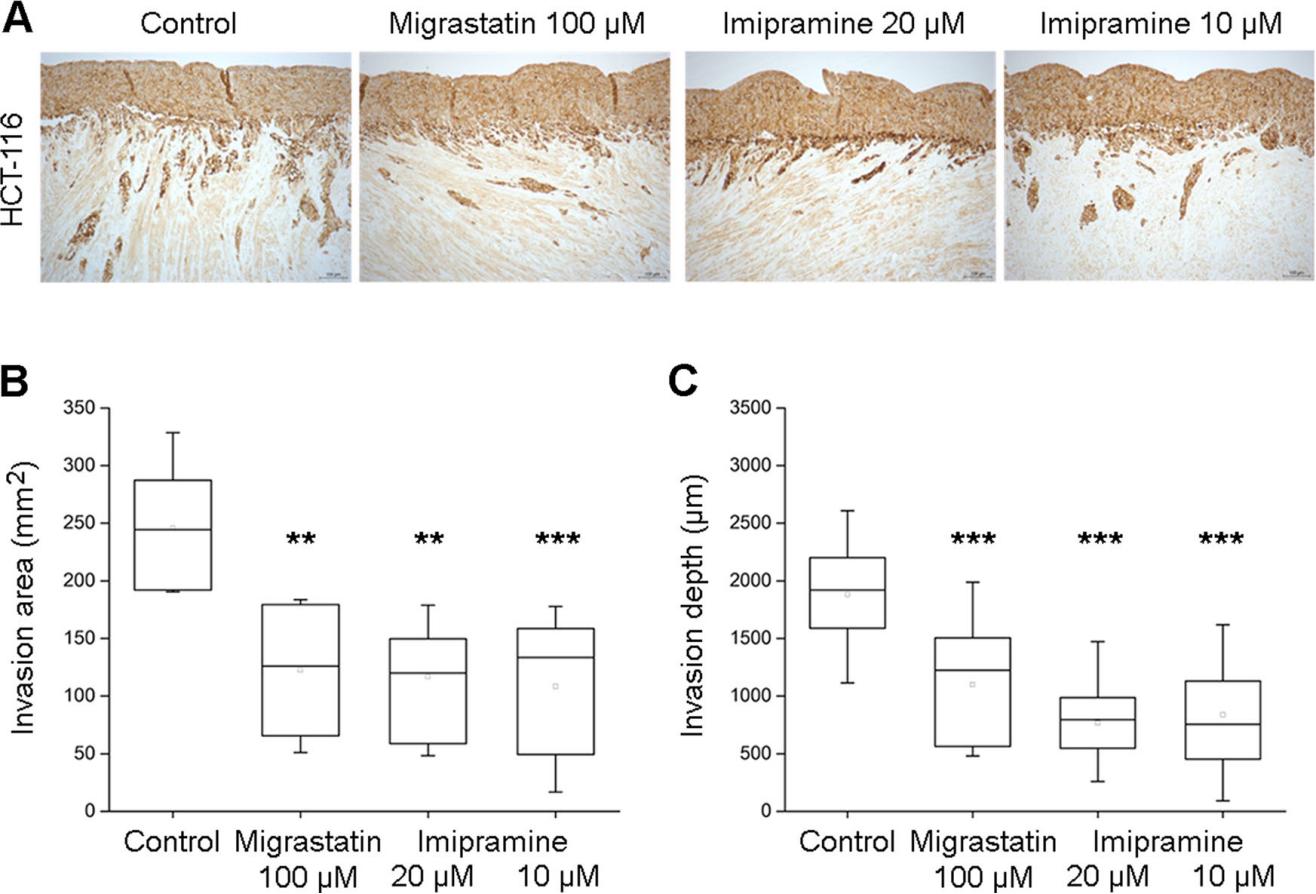
Myoma organotypic invasion model showing. **a** The effect of 100 µM migrastatin and imipramine (10 and 20 µM) on the HCT-116 colorectal cell line. Quantifiable effect on the invasion area (**b**) and on the invasion depth (**c**). **p < 0.001, ***p < 0.0001.

To determine whether the anti-invasive properties of imipramine could be translated into human tissue of stromal origin, a myoma organotypic model was used to test the invasion of HCT-116 colorectal cancer cells. As shown in Fig. 6, imipramine inhibited both the depth and the area of invasion of HCT-116 cells compared with control conditions and to a comparable extent as migrastatin.

### Imipramine inhibits the invasive and metastatic capacity of colorectal tumor cells in an in vivo model

**Fig. 7:**
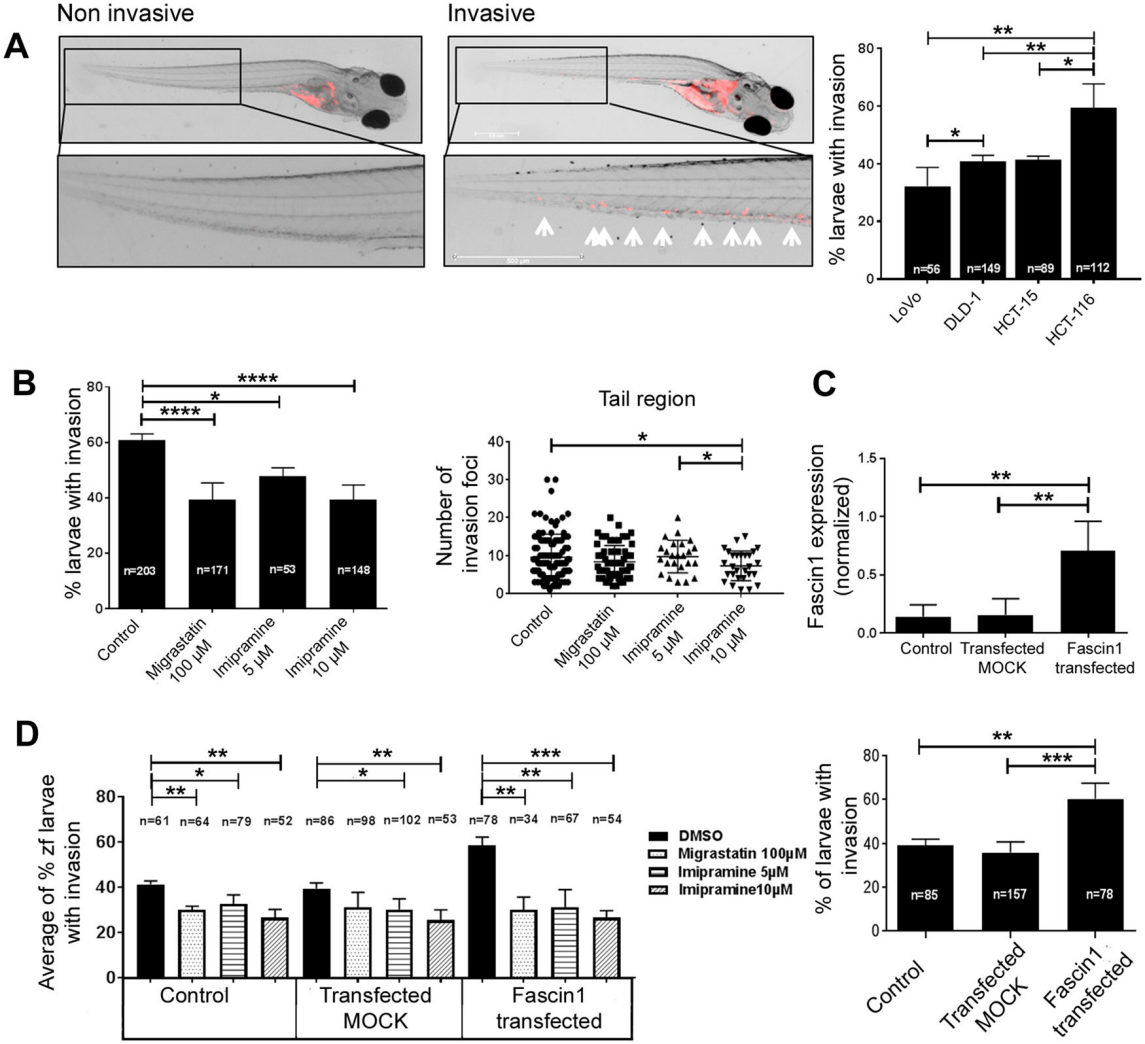
Zebrafish invasion assays 4 days post xenograft. **a** The images show the invasive and noninvasive cells in a zebrafish invasion model. The invasiveness of each cell line in this model is shown in the right panel. **b** HCT-116 cancer cells were treated with migrastatin and imipramine and then injected into zebrafish larvae. The effect of imipramine in diminishing the cell invasion percentage and the number of invasive cells is similar to that of migrastatin. **c** Fascin1 mRNA levels in transfected DLD-1 cells and cell invasion associated with transfection (lower panel). **d** Effect of drugs on the average percentage of zebrafish larvae invasion in fascin1-transfected DLD-1 cells. Data are shown as the mean ± SD, compared with the control, *p = 0.049–0.01; **p = 0.001–0.009; ***p = 0.0001–0.0009; ****p < 0.0001.

To undercover whether the anti-invasive properties of imipramine could be extrapolated to an animal system, the well-established zebrafish larvae invasion model was used, and a xenograft assay was performed (Fig. 7a). The vast majority of larvae were viable when treated at all concentrations; however, the combination of 20 µM imipramine and the injection of naive HCT-116 or transfected DLD-1 resulted in lethality for all larvae after the second day post xenograft (Supplementary Information S9). To check for a dose-dependent effect, 5 and 10 µM concentrations of imipramine were included in the following experiments. The percentage of invasion correlated with fascin1 mRNA expression; HCT-116 had the highest expression, and LoVo and DLD-1 cells had the lowest fascin1 expression and invasion percentage (Fig. 7a). For that reason, HCT-116 was selected as the cell line with the highest constitutive fascin1 expression, and fascin1-transfected DLD-1 cells were selected as the condition to test the effect of induced fascin1 expression. The fascin1-transfected cells increased the level of protein expression and the percentage of zebrafish larvae invasion (Fig. 7c).

**Fig. 8:**
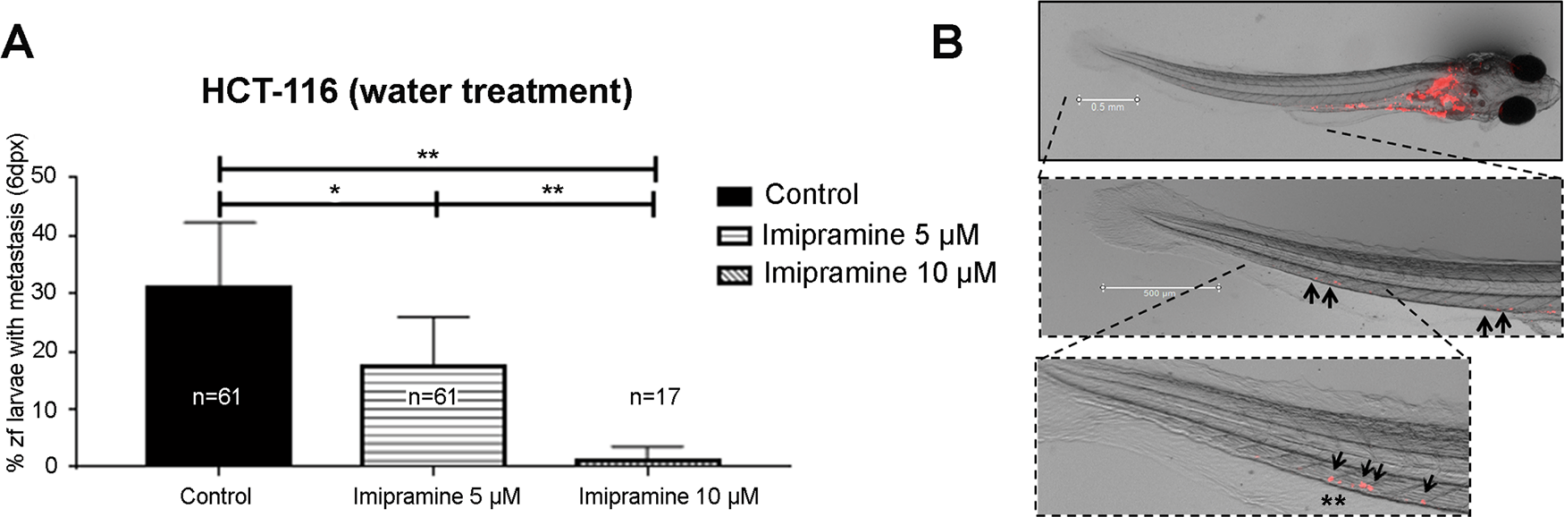
Antimetastatic potential of imipramine in zebrafish. **a** At day 6 post injection, larvae were examined to evaluate whether micrometastasis developed by invading native HCT-116 cells. **b** The evaluation criteria were the presence of human cancer cell colonies (arrows) outside the yolk sac. Invading single cells are indicated by asterisks (*). Data are shown as the mean ± SD, compared with the control, *p = 0.049–0.01; **p = 0.001–0.009.

Imipramine treatments of larvae exhibited a significantly lower percentage of invasion and a lower number of invasive HCT-116 cells than the control, similar to that observed for migrastatin (Fig. 7b). Similarly, E3 medium treated with imipramine and migrastatin (treatment after cell injection) also diminished fascin1-transfected DLD-1 cell invasion, and this effect was dose-dependent for imipramine (Fig. 7d). The same inhibitory effect was observed when tumor cells were treated prior to injection (Supplementary Information S10). When larvae were fed and kept alive 6 days post xenograft, micrometastasis developed from invading tumor cells. As presented in Fig. 8, HCT-116 colorectal cancer cells were treated with 5 and 10 µM imipramine, causing a significant decrease in the number of larvae with metastasis in a dose-dependent manner. When DLD-1 cells were transfected with the fascin1-GFP vector, a significant increase in larvae with metastasis was observed compared with the pGFP-N3 control vector (MOCK). This metastatic activity diminished when larvae were treated with the lowest imipramine dose with anti-invasive activity (Supplementary Information S11).

## Discussion

Our prior study demonstrated, using expression profiling, that fascin1 is overexpressed in SAC compared with CC (88.6% vs. 11.6%)^3^. The high frequency of KRAS or BRAF mutations confers SAC with an important resistance to current anti-epidermal growth factor receptor therapies^9,10^. Similarly, several authors have reported that fascin1 is overexpressed in triple negative/basal phenotype breast carcinoma^22–24^. Given the role of fascin1 in invasion and metastasis, we performed a bioinformatic search for in silico identification of chemical compounds blocking this protein.

We found that imipramine, a tricyclic antidepressant (TCA), gave a high score in the bioinformatics analysis, which was subsequently validated in vitro by Thermofluor, fluorescence titration experiments, and TEM. The application of imipramine in fascin1-overexpressing tumors is patent pending by the authors (no. 18382696.5). A previous study by Jahchan et al.^25^ demonstrated the antitumoral effects of imipramine in human small cell lung cancer and other neuroendocrine tumors implanted in mice. In a clinical setting, Sauer and Lang reported unexpected survival associated with imipramine treatment in a patient with metastatic lung cancer^26^, and the epidemiological study by Walker et al.^27^, which included 31,953 cancer cases from different locations and 61,591 matched controls, concluded that tricyclic antidepressants such as imipramine may have potential for the prevention of both colorectal cancer and glioma in a dose- and time-dependent fashion. The antidepressant effects of TCAs are thought to be due to an overall increase in serotonergic neurotransmission (https://www.drugbank.ca/drugs/DB00458); however, despite the aforementioned evidence, no clear molecular targets have been linked to the antitumoral activity of imipramine. Compelling evidence suggests that imipramine is able to cross the plasma membrane and inhibit intracellular targets. Indeed, CACO-2, a colorectal cancer cell line used to test drug intestinal absorption, was found to be permeable to imipramine (https://www.drugbank.ca/drugs/DB00458).

Furthermore, Kraft et al., by screening 1040 compounds using cultured fascin1-deficient mutant *Drosophila* neurons, whose neurite arbors manifest the “filigree” phenotype, identified imipramine as a fascin1 pathway blocker. Moreover, these authors demonstrated that single substitutions found in other antidepressants (desipramine, trimipramine, and clomipramine) suppress this anti-fascin1 pathway phenotype^28^. Nonetheless, none of these other antidepressants received a significant score in our in silico screening, thus suggesting a direct binding of imipramine to fascin1 and not to other proteins of its pathway. The functional enrichment analysis presented here is also suggestive of an effect of imipramine on the actin cytoskeleton because 3 out of 18 GO molecular functions associated with imipramine treatment are cytoskeleton-related. Despite this evidence, an additional off-target antitumoral effect of imipramine beyond fascin1 is also possible.

The relationship between neural markers and fascin1 overexpression was further confirmed by the fact that neuroblastoma cell lines have the highest fascin1 expression and that Munson et al.^29^ demonstrated that imipramine blue, an imipramine derivative, showed anti-invasion properties against malignant glioma cells in vitro and in vivo. As Kraft et al. pointed out, although the design of the study by Munson et al. was based on the inhibition of NADPH oxidase by imipramine blue, glioma cells treated in vitro showed a dramatic reorganization of their actin cytoskeleton, with marked loss of actin bundle-based protrusions and extensions^28,29^, which is consistent with our findings of a direct effect of imipramine causing loss of fascin1 function^28^.

Of note, in our study, the HCT-116 colorectal cell line was used for its highest fascin1 expression out of eight CRC cell lines. However, we cannot assure that the primary tumor for this cell line could be an SAC, as this information was not recorded when establishing tumor cell lines, and there is no CRC cell line typified as from SAC origin. Previous articles highlight that cancer cell lines maintain their morphological features and metastatic potential in zebrafish xenografts and further validate the chemosensitive profile of HCT-116 cells in zebrafish and mouse xenografts. The results in mouse xenografts closely matched with zebrafish xenografts^20,30^. It is worth noting that in our study, imipramine did not seem to be toxic to zebrafish at anti-invasive doses.

This study reports, for the first time, an antimigratory and anti-invasive effect of imipramine, an FDA-approved antidepressant oral agent, in colorectal tumor cells possibly due to anti-fascin1 activity, thus paving the way for a new molecular targeted treatment in SAC and other fascin1-overexpressing tumors.

## Supplementary information

Supplementary Information
