# Supplementary material for: New role of the antidepressant imipramine as a Fascin1 inhibitor in colorectal cancer cells
Source: Exp Mol Med. 2020 Feb 20;52(2):281–92. doi: 10.1038/s12276-020-0389-x (PMC7062870; doi:10.1038/s12276-020-0389-x)

Supplementary Information

Supplementary Information S1. Detailed description of the experimental protocols and tables.

Supplementary Information S2. Differential scanning fluorimetry profiles for fascin1 at different protein concentrations (A) and different concentrations of DMSO (B) showing that fascin1 unfolds as a monomeric protein with no effect of the protein concentration on the denaturation profile and with slight sensitivity to DMSO.

Supplementary Information S3. mRNA expression levels of fascin1 in eight colorectal cell lines using β-actin mRNA gene expression for data normalization.

Supplementary Information S4. Colorectal cancer cell line viability assay. The effect of migrastatin (upper panel) and imipramine (lower panel) on the cell viability of DLD-1, HCT-116, and SW-480 colorectal cell lines is shown.

Supplementary Information S5. Panther pie chart of the 18 differentially enriched functions. As indicated, actin binding (GO: 0003779), cytoskeletal protein binding (GO: 0008092), and structural constituent of cytoskeleton (GO: 0005200) were found among these 18 functions.

Supplementary Information S6. Cell migration assay for the three colorectal cancer cell lines after 7 h. (A) HCT-116, (B) DLD-1, (C) SW-480, and (D) migration were calculated with respect to the control conditions for a slope between 4 and 7 h (linear phase). * $p<0.05$, ** $p<0.01$.

Supplementary Information S7. Inhibition of the migration and invasive capacities by imipramine in transfected cells. A) Percentage of migration. B) Percentage of invasion. CRC HCT-116 cells were genetically knocked down for fascin1 with short interfering

siRNA. MOCK control HCT-116 cells were transfected with siRNA-A. CRC DLD-1 cells genetically overexpressed fascin1 with the fascin1-GFP vector. MOCK control DLD-1 cells were transfected with the pGFP-N3 control vector. Data are representative of two similar experiments with error bars, mean ± SD of duplicates. * $p<0.05$, ** $p<0.01$ compared with the MOCK condition; n.s.: nonsignificant.

Supplementary Information S8. Transwell invasion assays were performed to evaluate the HCT-116 cell invasive ability. (A) Invasion after treatment with migrastatin (100 µM) and imipramine (20 µM). Pictures were taken under an inverted phase contrast microscope. The magnification was ×200, and the scale bars =50 µM. (B) Quantification of the invasive cells by DO in a spectrophotometer at λ=560 nm. (C) Number of invasive cells using ImageJ software. Data are presented as the mean ± SD compared with the control.

Supplementary Information S9. Survival curves of the zebrafish larvae when treated with the compounds in E3 medium (A) and when they were injected with HCT-116 or transfected DLD-1 tumor cell lines (B).

Supplementary Information S10. Invasion assay with colorectal tumor cells treated with the drugs prior to injection into the zebrafish larvae. (A) DLD-1, (B) HCT-116, (C) Anti-invasive effect of the drugs on fascin1-transfected DLD-1 cells.

Supplementary Information S11. Effect of imipramine on the development of micrometastasis caused by transfected DLD-1 cells overexpressing fascin1 in zebrafish. (A) The imipramine dose was set as 5 µM, as it was the lowest dose showing anti-invasive properties. (B) Transfection quantitation of fascin1 mRNA expression in transfected DLD-1 cells with the pGFP-N3 control vector (MOCK) and the fascin1-GFP vector.

Supplementary Information S1. Detailed description of experimental protocols and tables.

Thermofluor and fluorescence titration

Thermofluor was performed using a Biorad C1000 Touch Thermal Cycler CFX96 RT-PCR system in a 96-well format. A total of 25 µL reaction mixtures were set up containing 2 µM fascin1 (cat. no. 8411-02, Hypermol, Bielefeld, Germany) in 20 mM Hepes, 150 mM NaCl, 1mM DTT, and 5% sucrose at pH 7.4, in the presence of SYPRO Orange (1,000-fold dilution from the commercial stock [Invitrogen]). The indicated compound, prepared at 10 mM in 100% DMSO, was added to each well to a final concentration of 1 mM and 10% DMSO. Included in the 96-well plates were three replicates per compound, together with six internal controls, containing only free protein in 10% DMSO. The PCR plates were covered and subsequently shaken, centrifuged, incubated for 2 min at 20ºC inside the RT-PCR machine, and heated from 20ºC to 100ºC at a 1ºC/min scan rate. Fascin1 was extensively dialyzed against the appropriate buffer prior to the titration experiment, and its concentration was determined by measuring the absorbance at 280 nm using an extinction coefficient of 67840 $cm^{-1} \cdot M^{-1}$. Fluorescence titration experiments were performed in a Cary Eclipse spectrofluorometer (Varian Inc.). A 15 µM fascin1 solution, kindly provided by Dr. Steven Almo from Albert Einstein College of Medicine (New York, USA), was titrated by adding increasing volumes of concentrated solutions of imipramine. Emission spectra were recorded between 307 and 500 nm at 25ºC in 10% DMSO, 100 mM NaCl, 20 mM Hepes, and pH 7.4, with the excitation wavelength fixed at 280 nm. Binding isotherms were generated and fitted using ORIGIN 7.0 (Microcal Inc.) to a one-site equilibrium binding model, according to the following equation:

$$F = F_f + (F_b - F_f) \cdot \frac{(P_T + L_T + K_d) - \sqrt{(P_T + L_T + K_d)^2 - 4 \cdot P_T \cdot L_T}}{2 \cdot P_T}$$

where Ff and Fb are the fluorescence signal of free and bound fascin1 and PT and LT are the total protein and ligand concentration, respectively, at each addition point.

F-actin bundling assay

Rabbit muscle actin was induced to polymerize to F-actin using 250 µL of general actin buffer, leaving it on ice for 30 min, and adding 25 µL of actin polymerization buffer at room temperature (RT) for 1 h. Test protein, that is, human recombinant fascin1 (Hypermol), was centrifuged (10.000 *g*, 1 h, 4ºC) to obtain the test protein stock, which was diluted at 1 µM and then incubated with imipramine (100 µM) and DMSO (4%) for 30 min at RT. After that, all samples and controls were subsequently incubated with F-actin (10 µM) for another 30 min at RT. F-actin bundling activity was determined performing a low-speed centrifugation after incubation (10.000 *g*, 1 h, RT). Comparable amounts of supernatant and pellet fractions were analyzed by densitometric scanning of Coomassie blue-stained SDS/polyacrylamide gels. The intensities of protein bands in Coomassie-stained gels were measured, and the relative F-acting bundling activity was calculated using ImageJ software.

Transmission electron microscopy

In the control condition, purified actin (21 µM) was polymerized according to the protocol from the Actin Binding Protein Biochem Kit™ Muscle Actin and then incubated with human recombinant fascin1 (molar ratio 1:1) for 30 min at RT. Fascin1 was previously incubated for 2 h with 100 µM migrastatin and 10 µM imipramine. The samples were directly adsorbed onto 200 mesh copper grids for 30 s, blotted to remove excess solution, washed twice with distilled water, and negatively stained with 1%

(w/v) uranyl acetate for 30 s, blotted and dried again. The TEM study of actin filaments and fascin1-actin bundles was performed on a PHILIPS TECNAI 12 transmission electron microscope (Japan) at an accelerating voltage of 80 kV and a magnification up to 135.000 X. Images were captured on a coupled device camera (Megaview III). The numbers of filaments per bundle were counted manually in 25 pictures/condition and statistically analyzed (Kruskal–Wallis test).

Fascin1 mRNA expression assessment by quantitative PCR

RNA was extracted by treating cell line pellets (around 200,000 cells) with 700 µL of Qiazol (Qiagen ref: 1023537) and by adding 140 µL of chloroform and centrifuging at 12,000 *g* for 15 min at 4ºC. The aqueous phase containing 350 µL was then subjected to automatic total RNA extraction using the Qiacube equipment and the miRNeasy Mini Kit (ref: 217004), both provided by Qiagen (Hilden, Germany). cDNA was obtained using the Maxima First Strand cDNA Synthesis Kit by Thermo Fisher (Fisher Scientific, cat. no. K1671, Madrid, Spain) following the manufacturer information. A total of five microliters of 1:5 diluted cDNA was added to the qPCR reaction containing 12.5 µL 2X QuantiTect SYBR Green PCR Kit (ref: 204145) by Qiagen and 300 nm of each primer in a total volume of 25 µL. qPCR was performed on a 7500F real-time PCR system by Applied Biosystems (Foster City, California, USA) following the standard protocol: 50ºC 2 min, 95ºC 10 min, 40 cycles of 95ºC 15 sec, 60ºC 1 min and a melt curve stage consisting of 95ºC 15 sec, 60ºC 1 min, 95ºC 30 sec, and 60ºC 30 sec. The relative quantitation was obtained by the 2-ΔCt method using β-actin as housekeeping gene. The amounts of mRNA are given as number of copies per million of copies of β-actin. Primers of β-actin used for FSCN1 quantitation are shown below:

Primer sequences used for quantitative PCR validation

**Gene**	**Primer name**	**Sequence (5´-3´)**	**Fragment size (bp)**
FSCN1 (ENSG00000075618)	115F	TCCACGCGCCAGGGTATGGAC	121
	116R	ACTTGCCCGTGTGGGTACGG	
β-ACTIN (ENSG00000075624)	b-a-blgF	GAGCTACGAGCTGCCTGACG	120
	b-a-blgR	GTAGTTTCGTGGATGCCACAG	

Cell viability assay

Exponentially growing cells were plated in triplicate in flat-bottomed, 96-well plates (Nunc, Roskilde, Denmark) at 1,500 cells/well. On the day after, drugs were added in serial dilution from 500 nm to 300 µM of migrastatin and imipramine compounds. Control wells contained medium without drug plus 0.1% DMSO (drug carrier). Plates were incubated at 37ºC for three days in a humidified 5% CO_2 incubator and assayed for cell viability. Tetrazolium dissolved in Dulbecco's phosphate-buffered saline (PBS), pH 7.2, at 1.9 mg/mL was added to the cells (30 µl/well). After incubation at 37°C for 4 h, the medium was aspirated. The formazan crystals were dissolved in 200 µL DMSO for 30 min, and the absorbance was read at 570 nm in a microtiter plate reader. Results were calculated as cell viability (%) = average OD of wells/average OD of control wells.

RNA labeling, microarray hybridization, and function enrichment analysis

To have a general overview of enriched functions associated with the treatment of colorectal cancer tumor cells, HCT-116 cells were treated with DMSO or imipramine. Total RNA was isolated using the miRNeasy Mini Kit (Qiagen, Hilden, Germany) and quantitated on a NanoDrop 2000 (Thermo Fisher Scientific, Waltham, Massachusetts). RNA quality was examined on an Agilent 2100 Bioanalyzer (Agilent Technologies, Palo Alto, California) using the RNA 6000 Nano Kit. All the samples studied had RIN (RNA Integrity Number) =10. RNA samples were labeled using Agilent Two-Color Quick Amp Labelling and RNA Spike-In kits, according to the manufacturers' protocol. Experimental samples were labeled with cyanine 5-CTP and used as tests. The Universal Human Reference RNA (Agilent Technologies) was labeled with cyanine 3-CTP and used as reference. The labeled cRNAs were mixed together and hybridized onto SurePrint G3 Human Gene Expression v3 8x60K Microarrays, targeting 26,083 Entrez Gene RNAs and 30,606 long noncoding RNAs (lncRNAs), using the Agilent Gene Expression Hybridization Kit. Data sets were extracted using the Agilent Feature Extraction software. The normalized expression values were obtained using the function NormalizaWithinArrays with the Lowess method. Probes were collapsed at the gene level using the Avereps function, and the differences between DMSO and imipramine were expressed as log2FC averages. Only one experiment was performed per condition, and thus, no p values were obtained. Enriched functions associated with the gene list were obtained using Gene Ontology category of "molecular function" and Panther Classification System (accessible at http://www.pantherdb.org).

Data sets deposited at the Gene Expression Omnibus (GEO) database under accession number GSE125169 at https://www.ncbi.nlm.nih.gov/geo/query/acc.cgi?acc=GSE125169.

Immunofluorescence

After cells reached 100% confluence, serum-supplemented medium was removed and replaced with fresh serum-free medium for 24 hours. Artificial wounding was performed by transversally dragging a sterilized razor blade on the central area of the coverslips. Coverslips (Thermo Fisher, Waltham, Massachusetts) were then placed in a six-well plate with 2 mL serum-free DMEM and 100 µM migrastatin, 20 µM imipramine, 10 ng/ml EGF, or 50 µM MEK inhibitor PD98059 (MEKi) (both from Sigma-Aldrich, St. Louis, Missouri, USA) for 24 h. Cells were then fixed with Bouin (for fascin1 protein) or 4% formaldehyde (for actin protein) and subsequently permeabilized in a 0.3% Triton X-100/PBS solution and then exposed to blocking buffer for 30 min. Samples were incubated for 1 h with anti-fascin1 antibody (1/250) (55K-2 clone; Santa Cruz Biotechnology, Heidelberg, Germany; Santa Cruz Biotechnology Cat# sc-21743, RRID: AB_627580) or anti-β-actin antibody (1/1000) (Sigma-Aldrich Cat# A5441, RRID: AB_476744, Sigma-Aldrich, St. Louis, Missouri, USA) in a wet chamber. Appropriate fluorescent-labeled primary antibodies were incubated later with Alexa fluor 488-conjugated anti-mouse IgG (from donkey) (1/400) (Molecular Probes Cat# A-21202, RRID: AB_141607) or Alexa fluor 594-labeled phalloidin (1/1000) (Thermo Fisher Scientific Cat# A12381, RRID: AB_2315633) and Hoechst 33258 (Thermo Fisher Scientific Cat# H3569, RRID: AB_2651133) for 30 min at room temperature and darkness. Samples were examined, and representative images were taken with a confocal microscope (LSM 510 META from ZEISS, Jena, Germany).

Transfection assay

To quantify the effect of imipramine upon fascin1-induced and -silenced expression in colorectal cancer cells, transfection assays were performed prior testing the *in vitro*

migration capacities and in the zebrafish invasion model. The human CRC cells were genetically overexpressed (DLD-1) and knocked down (HCT-116) for fascin1. HCT-116 (4 10^5 cells/well) and DLD-1 (9 10^5 cells/well) were seeded in six-well plates and then, cultured overnight, grown to 50%–70% confluence. In the case of DLD-1, pGFPN3 control vector or fascin1-GFP vector (kindly provided by Dr. Milind Valdya from the Advanced Centre for Treatment Research and Education in Cancer, Maharashtra, India) was used. HCT-116 cells were transfected with fascin1, short interfering siRNA or control siRNA-A (Santa Cruz Biotechnology, Heidelberg, Germany). Transfections were conducted using lipofectamine 2000 (Thermo Fisher, Waltham, Massachusetts, USA) according to the manufacturer's protocols. The cells were then washed with PBS before adding 800 µL standard medium without antibiotics. The final amount of nucleic acids used for transfection was 1 µg and 0.05 µmoles per well for DLD-1 and HCT-116 cells, respectively. The nucleic acids were diluted with OptiMEM I Reduced Serum Medium (Invitrogen, Carlsbad, California, USA). Equal volume of OptiMEM I medium (100 µL) was used to dilute lipofectamine 2000 and nucleic acids and then incubated for 5 min at room temperature. Mixture was added drop by drop to the lipofectamine-based liposome dilution and incubated at room temperature for additional 30 min. After incubation, the nucleic acids/liposome complexes were added to the cells and incubated for additional 6 hours. After transfection, the residual nucleic acids/liposome complexes were washed off, and the cells were replenished with standard medium and incubated up to 72 h. Fascin1 mRNA expression was checked with cell lysates by qPCR as described above.

Chemical treatment

HCT-116 and fascin1-transfected DLD-1 cells were treated for 24 h with 0.1% DMSO, 100 µM migrastatin, and 20 µM imipramine. For treatment larvae with drugs, zebrafish

embryos xenografted with HCT-116 and fascin1-transfected DLD-1 cell lines were transferred into 24-well plates and were treated, by bath immersion, with E3 medium (5 mM NaCl, 0.33 mM KCl, 0.33 mM $CaCl_2$, 0.33 mM $MgSO_4$, 0.1% methylene blue [Sigma]) containing 100 µM migrastatin or imipramine at 5 µM or 10 µM diluted in DMSO for 96 h. Fresh drug was added every 24 h. The number of individuals is shown in the graph bars.

End Notes

Patent applications

The following patent related to the findings presented in this manuscript has been submitted: P. Conesa-Zamora, H. Pérez-Sánchez, I. Luque-Fernández, S. Montoro-García, B. Alburquerque-González, P. Campioni-Rodrigues, J. García-Solano, A. Bernabé-García, F. J. Nicolás-Villaescusa, M. Bernabé-García, M. L. Cayuela-Fuentes, J. Ruiz-Sanz, J. C. Martínez-Herrerías, T. Salo, "Imipramine for use as inhibitor of fascin1 overexpression," EP18382696.5 (2018). Patent Pending.

Tables

Table S1. Thermal shift assay results

**Compound**	**Conc (mM)**	**$T_{m, FAM}$ (°C)**	**$\Delta T_{m, FAM}$ (°C)**	**$T_{m, HEX}$ (°C)**	**$\Delta T_{m, HEX}$ (°C)**	**$T_{m, TRed}$ (°C)**	**$\Delta T_{m, TRed}$ (°C)**
Free fascin1	-	55,7±0,5	-	56,0±0,0	-	56,2±0,6	-
Imipramine	1	58,3±0,6	2,7±1,1	58,0±0,0	2,0±0,0	58,0±0,0	1,8±0,6

Conc (concentration), T_m (melting temperature), ΔT_m (variation of melting temperature)

Table S2. Lamellipodium protrusion number in HCT-116 cells in the different treatment conditions

	**Control**	**Migrastatin 100 µM**	**Imipramine 20 µM**	**EGF 10 ng/mL**	**PD98059 50 µM**
Lamellipodium number	9 ± 1.5	2 ± 2	0.6 ± 0.8	10.4 ± 1.5	1 ± 1
P value*		**0,0001**	**3,305 E^{-06}**	0,1401	**5,9241E^{-06}**

EGF (epidermal growth factor)

Table S3. Percentage of migration in DLD-1, SW-480, and HCT-116 in control and the different treatment conditions

**Cell line**	**Treatment**	**Migration percentage**	**Standard deviation**
DLD-1	DMSO 0.1%	100	0
	Imipramine 20 µM	23.21	12.21
	Migrastatin 100 µM	67.91	5.19
SW-480	DMSO 0.1%	100	0
	Imipramine 20 µM	77.52	22.35
	Migrastatin 100 µM	70.34	18.04
HCT-116	DMSO 0.1%	100	0
	Imipramine 20 µM	43.25	6.82
	Migrastatin 100 µM	44.59	6.14

Supplementary Information S2

A

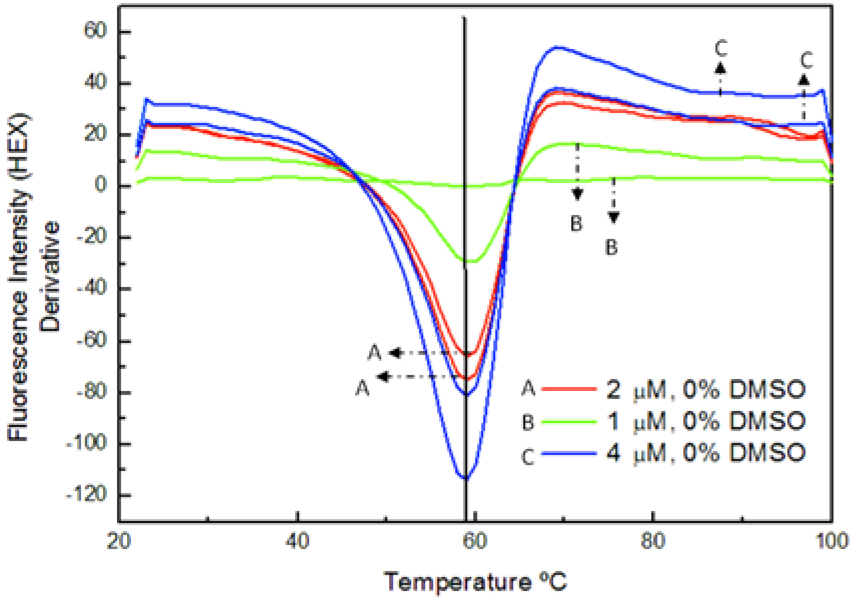

B

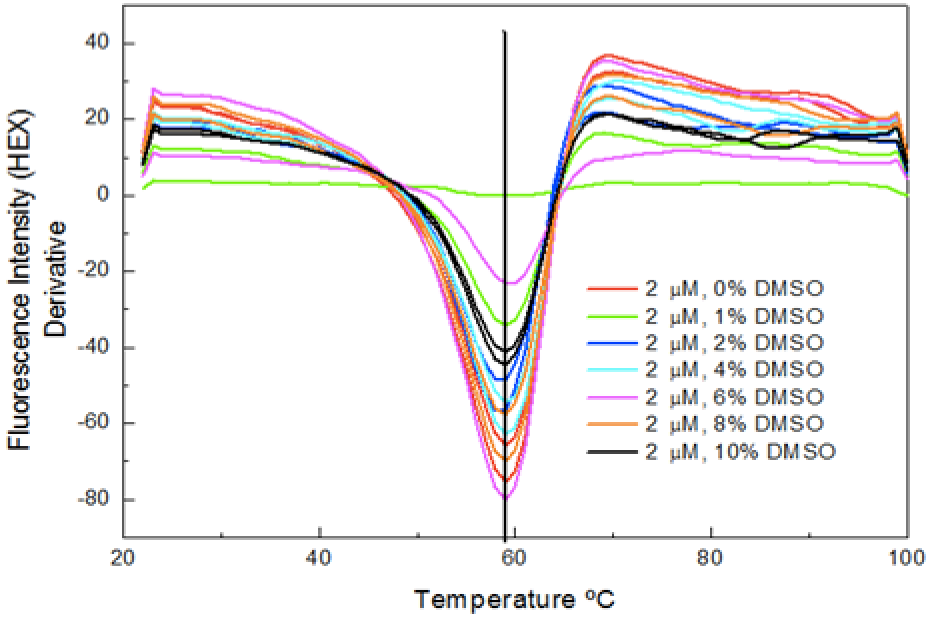

Supplementary Information S3

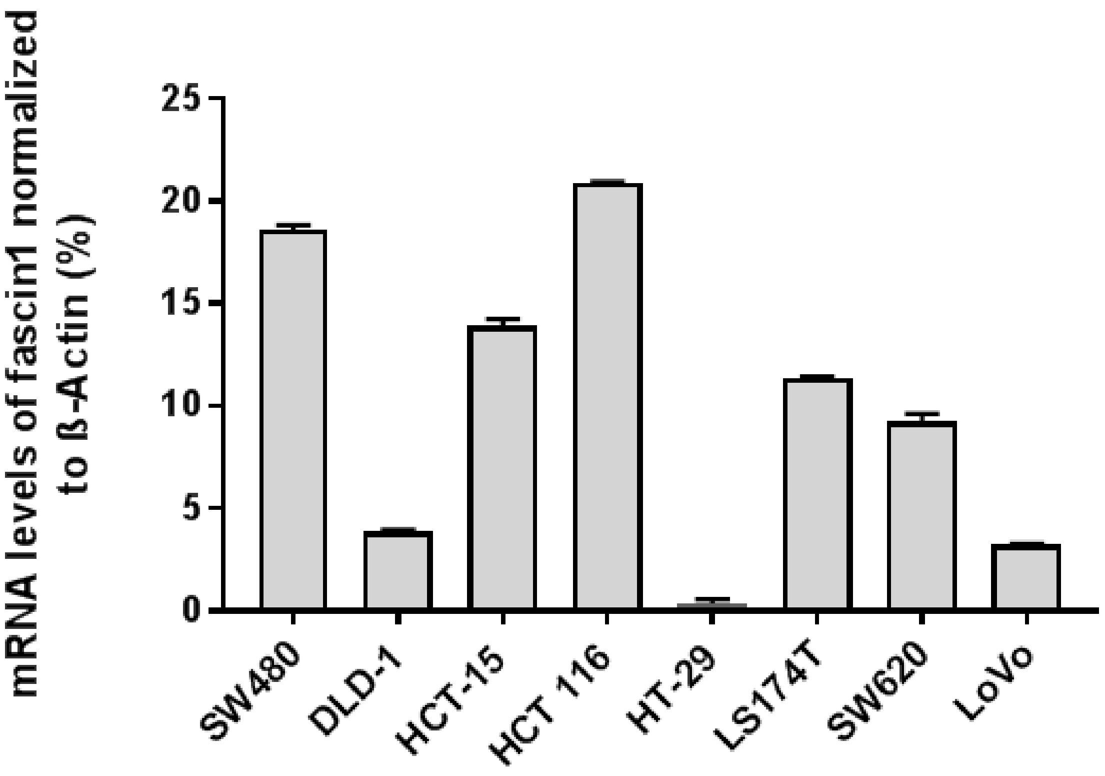

Supplementary Information S4

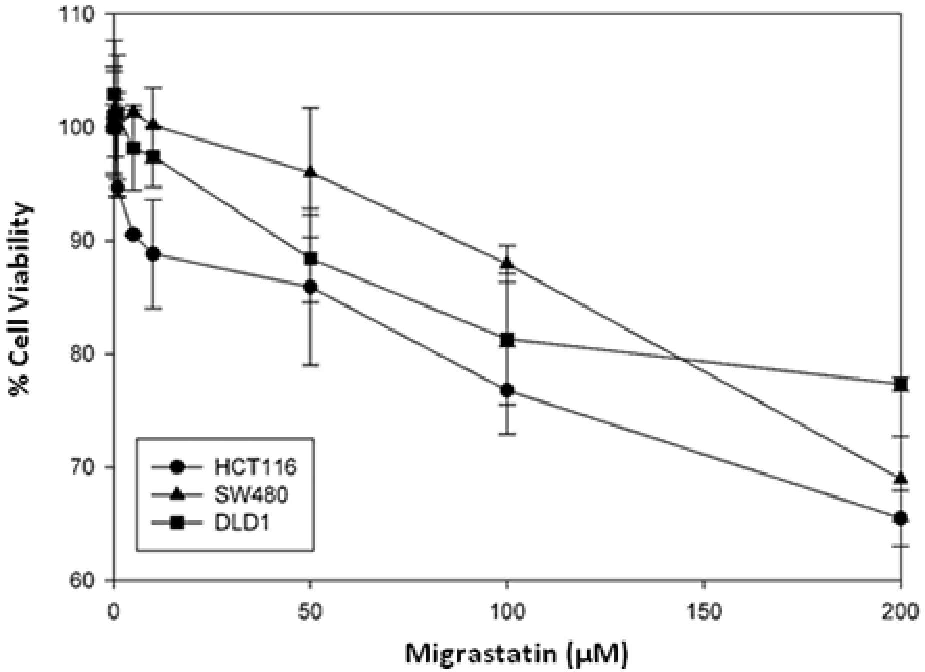

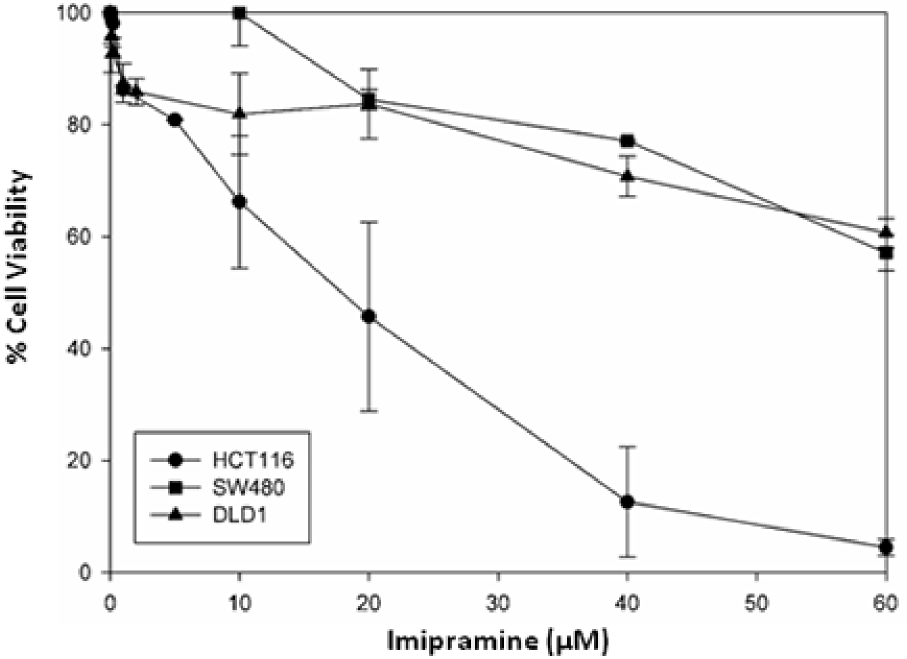

Supplementary Information S5

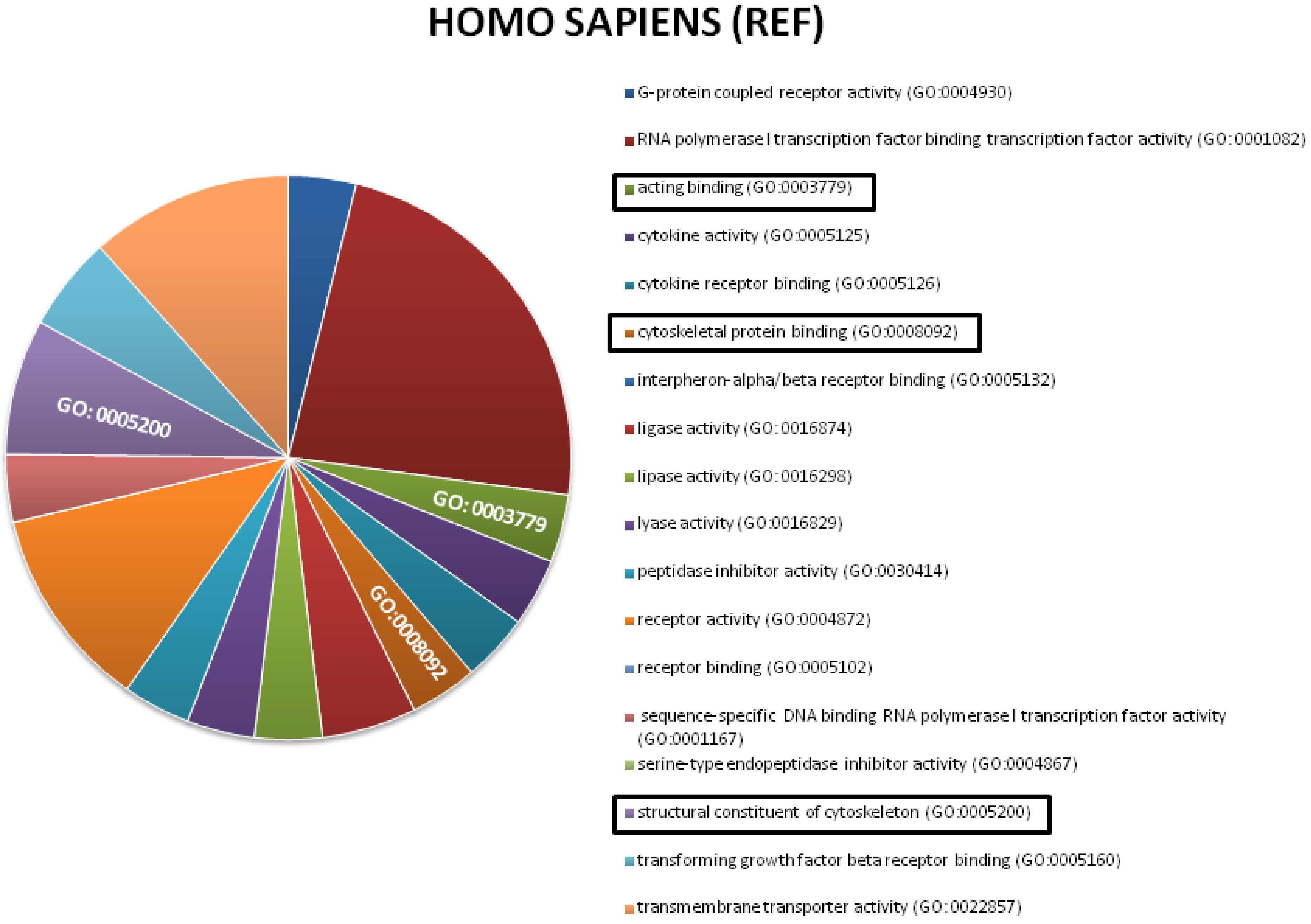

Supplementary Information S6

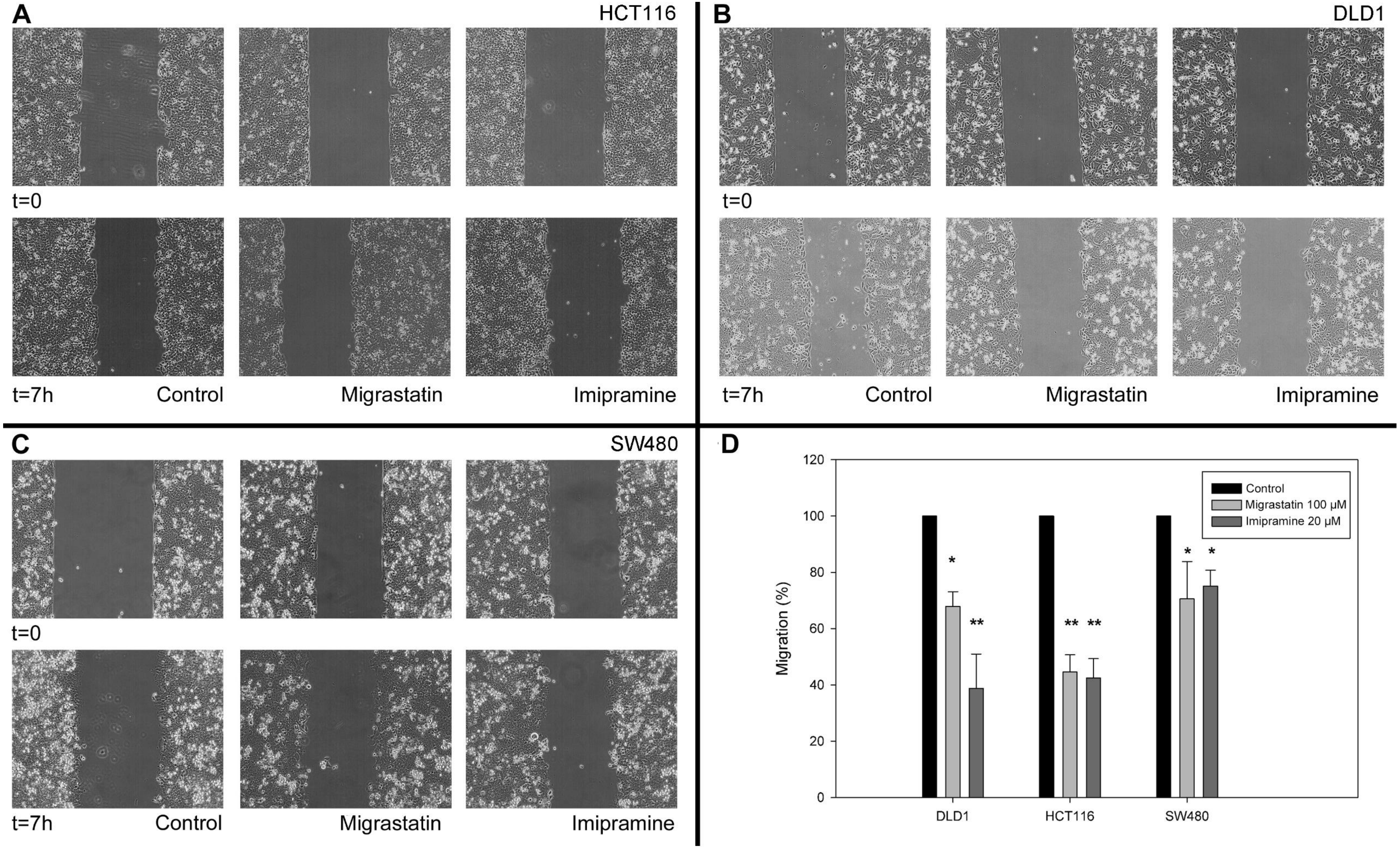

Supplementary Information S7

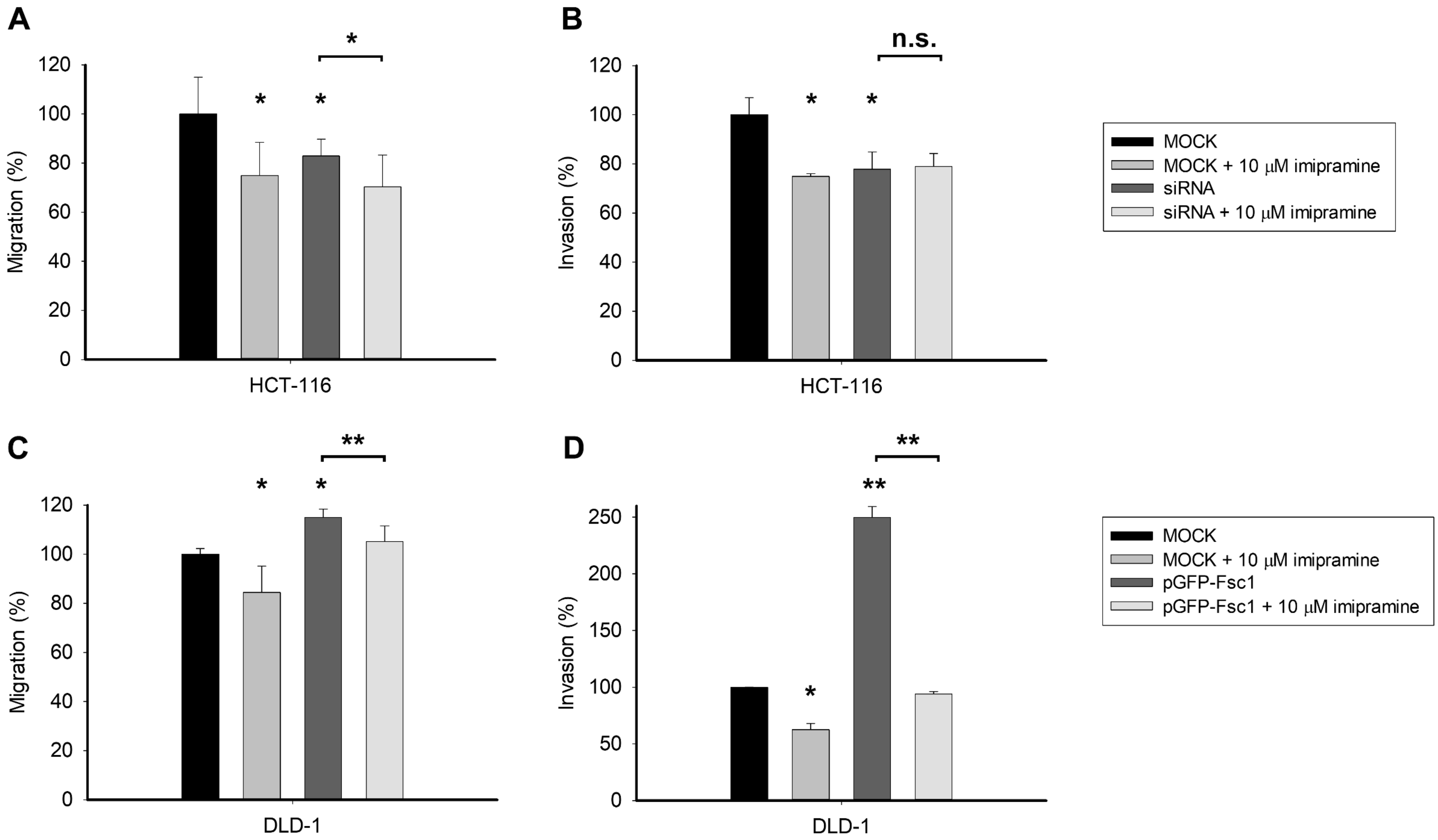

Supplementary Information S8

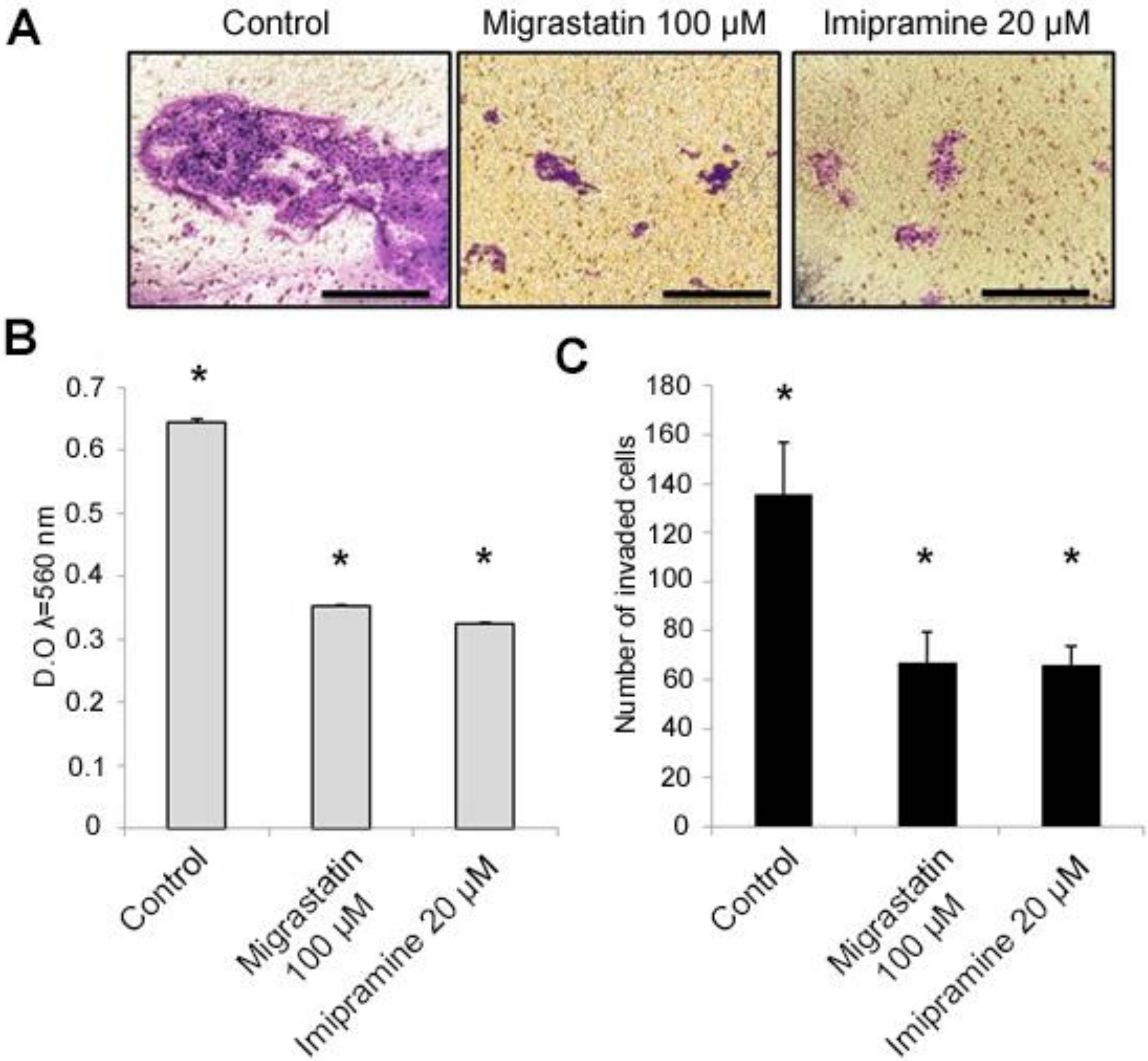

Supplementary Information S9

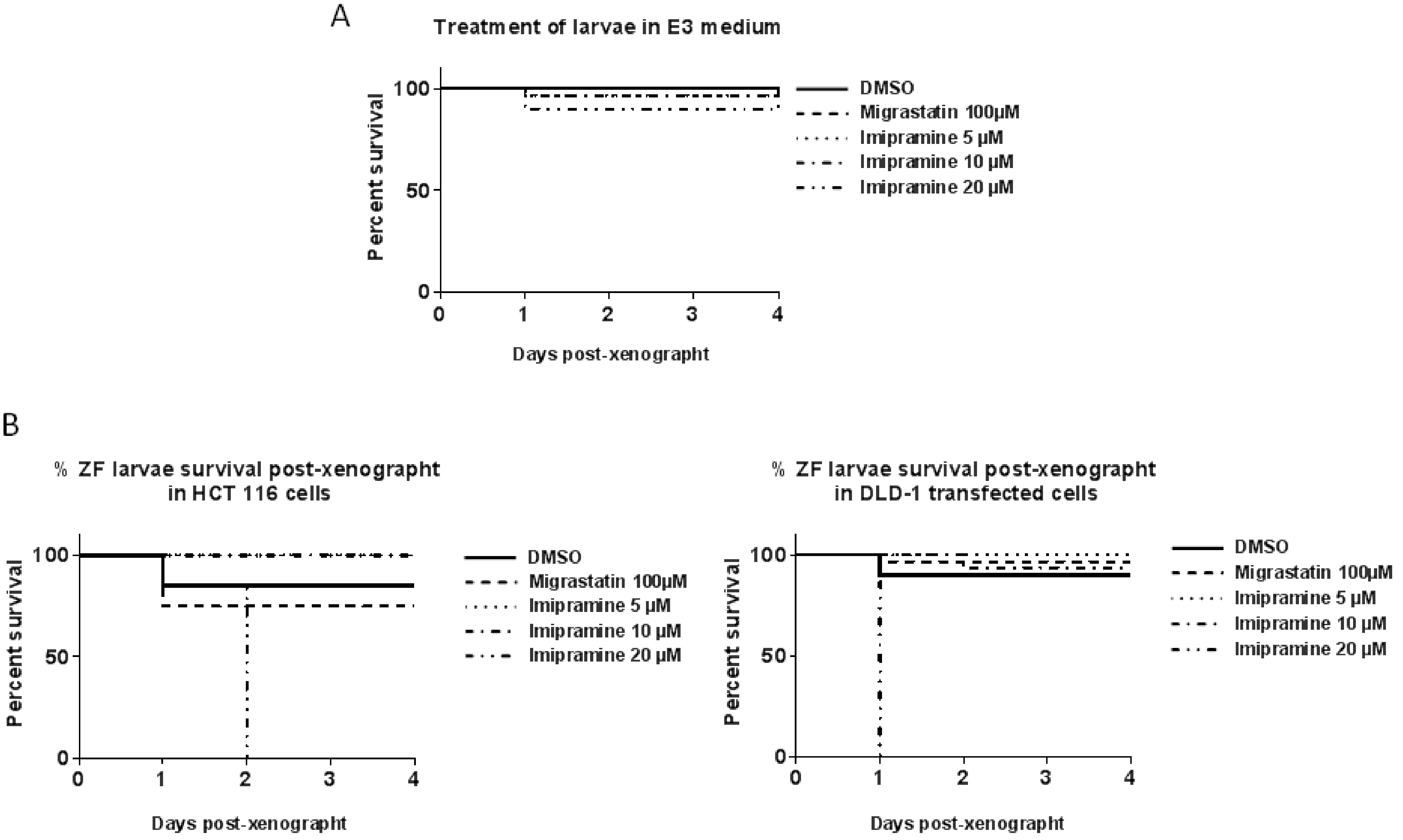

Supplementary Information S10

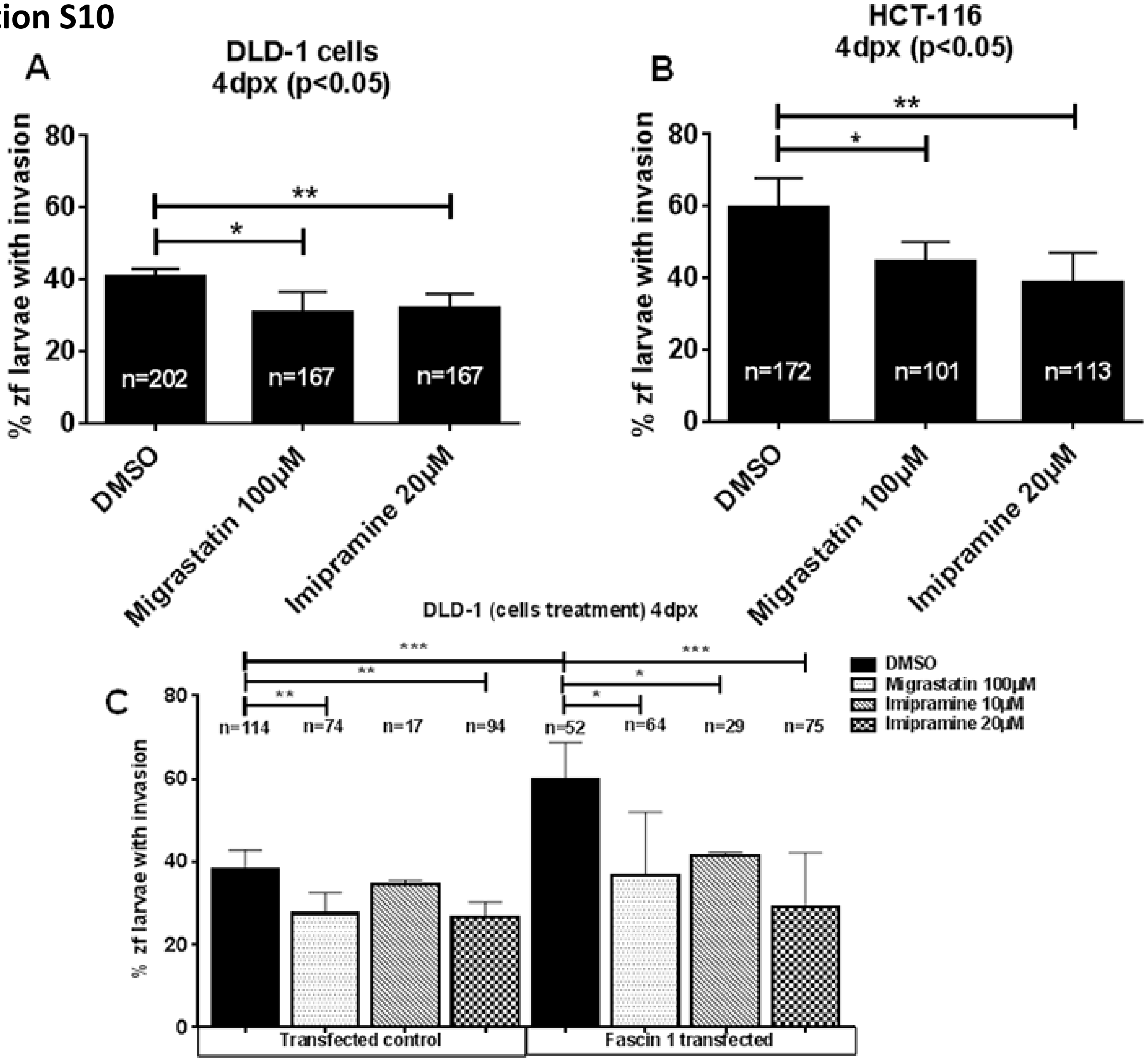

Supplementary Information S11

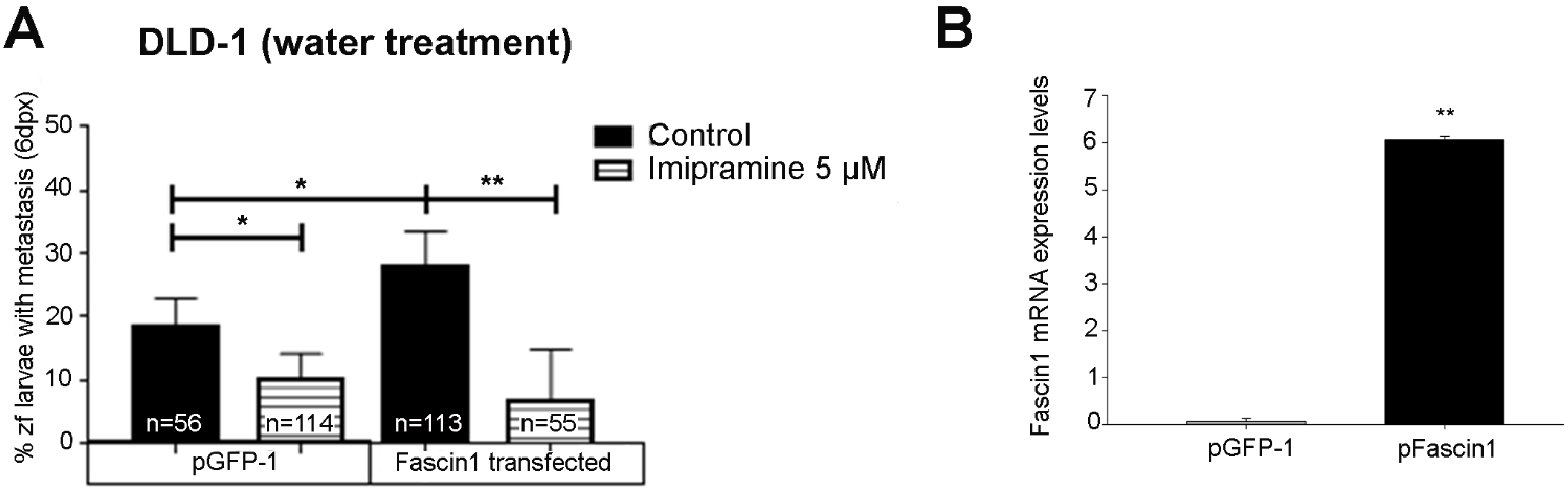

Supplement: Supplementary file 1 — Supplementary Information [file 12276_2020_389_MOESM1_ESM.pdf]
